# Supplementary material for: Associations of IGF2 and DRD2 polymorphisms with laying traits in Muscovy duck
Source: PeerJ. 2017 Nov 23;5:e4083. doi: 10.7717/peerj.4083 (PMC5702507; doi:10.7717/peerj.4083)
Supplement: Supplemental Information 1 — Table S1. Fifteen pairs of primers used in this study. Table S2. Raw data of association at IGF2 gene. Table S3. Raw data of association at DRD2 gene. Table S4. Raw data of association at DRD2 gene. [file peerj-05-4083-s001.doc]

**Associations of *IGF2* and *DRD2* polymorphisms with laying traits in Muscovy duck**

Qiao Ye^1, 2^ , Jiguo Xu^1, 2^ , Xinfeng Gao^1, 2^, Hongjia Ouyang^1, 2^, Wei Luo^1, 2^, and Qinghua Nie^1, 2^

^1^National-Local Joint Engineering Research Center for Livestock Breeding, Collegeof Animal Science, South China Agricultural University, Guangzhou 510642, Guangdong, China

^2^Key Lab of Chicken Genetics, Breeding and Reproduction, Ministry of Agriculture and Guangdong Provincial Key Lab of Agro-animal Genomics and Molecular Breeding, Guangzhou 510642, Guangdong, China

Corresponding author: Q. H. Nie. E-mail: nqinghua@scau.edu.cn.

**Supplementary table S1 Fifteen pairs of primers used in this study.**

| Primer Name^1^ | Sequence (5' to 3') | Product size (bp) | Tm (℃) | Purpose |
| --- | --- | --- | --- | --- |
| IGF2-CDS | F:CAGTTCGTCTGTGGGGACA | 438 | 58.1 | cDNA cloning |
|  | R:CCGCTGGGAGCTCTTCTT |  |  |  |
| DRD2-CDS | F:CCTATGGCTTGAAGGGTG | 1 451 | 53.9 | cDNA cloning |
|  | R:CGTGAATGGCATTGGATT |  |  |  |
| β-actin-duck | F:ACACCTTCTACAATGAGC | 190 | 50.5 | Internal control |
|  | R:ATACCAGTGGTACGACCA |  |  |  |
| IGF2-Q | F:GGCTCTGCTGGAAACCTA | 152 | 53.7 | RT-qPCR |
|  | R:TCTTCTTCTGCCACACGT |  |  |  |
| DRD2-Q | F:GGCAGCCCTGGGAAACTA | 226 | 52.2 | RT-qPCR |
|  | R:TGTGGGTGATGAAGAATGG |  |  |  |
| IGF2-P1 | F:CCAGACTGAAGGAGGAAT | 1 403 | 53.2 | SNPs detection |
|  | R:CCAAGGTCACAGGGGAAG |  |  |  |
| IGF2-P2 | F:GGCTTGGACACCCTAAAA | 1 310 | 55.3 | SNPs detection |
|  | R:TCCCCACAGACGAACTGC |  |  |  |
| DRD2-P1 | F:TACGGCACAATAGGCTGAG | 738 | 52.8 | SNPs detection |
|  | R:CAGGCAACAACCAAGTGA |  |  |  |
| DRD2-P2 | F:CAGTTAGCAGACAGGGAATG | 621 | 53.8 | SNPs detection |
|  | R:ACCAGAGTTGCCACCAAG |  |  |  |
| DRD2-P3 | F:CCTCTGGCTCTTCATCAC | 1 081 | 51.4 | SNPs detection |
|  | R:CGAGTATTTGGCTTTCACA |  |  |  |
| DRD2-P4 | F:TACATAAGTTACCGCATAGTTCC | 844 | 55.4 | SNPs detection |
|  | R:GCAGCAGGCAGCAATCT |  |  |  |
| DRD2-P5 | F:CGCAAAGTGGTGAGTGT | 602 | 49.4 | SNPs detection |
|  | R:GGGAGCAGCAATGAATA |  |  |  |
| IGF2-SNP | F:GCCAGACTGAAGGAGGAAT | 906 | 55.1 | Genotyping |
|  | R:GAAACCCAGCACAACCCT |  |  |  |
| DRD2-SNP1 | F:AATCCTTGCCTAGTGCC | 788 | 52.3 | Genotyping |
|  | R:AACCTGGTGTCCTTCCA |  |  |  |
| DRD2-SNP2 | F:CATCATTTGCGACCTTTAGT | 705 | 55.3 | Genotyping |
|  | R:AATGCCTTGAAGTCTTTCCT |  |  |  |

**Notes.**

^1^ Primer IGF2-CDS and DRD2-CDS are used for gene cDNA cloning; Prime β-actin-duck, IGF2-Q and DRD2-Q are used for RT-qPCR; Primer IGF2-P1, IGF2-P2, DRD2-P1, DRD2-P2, DRD2-P3, DRD2-P4 and DRD2-P5 are used for gene SNPs detection; Primer IGF2-SNP, DRD2-SNP1 and DRD2-SNP2 are used for gene SNPs genotyping.

**Supplementary table S2 Raw data of association at IGF2 gene**

| CAGE | cx_wb | FEA) | E59W | E300D | A-1864G | C-1704G |
| --- | --- | --- | --- | --- | --- | --- |
| 1801 | 17736 | 252 | 115 | 45 | AG | GG |
| 0029 | 13430 | 343 | 50 | 0 | AG | CG |
| 0057 | 15392 | 289 | 44 | 12 | GG | CG |
| 0060 | 14633 | 293 | 65 | 8 | AG | CG |
| 0141 | 13743 | 288 | 47 | 9 | GG | CC |
| 0142 | 13674 | 289 | 107 | 12 | AA | GG |
| 0143 | 10870 | 310 | 37 | 0 | AG | CG |
| 0145 | 10254 | 293 | 33 | 6 | AG | CG |
| 0146 | 14985 | 262 | 139 | 37 | AG | CG |
| 0147 | 12395 | 282 | 43 | 18 | GG | CC |
| 0148 | 12085 | 267 | 83 | 31 | AA | GG |
| 0149 | 13687 | 279 | 70 | 22 | AG | CG |
| 0150 | 15596 | 289 | 48 | 5 | AA | GG |
| 0151 | 14867 | 256 | 95 | 43 | AA | GG |
| 0152 | 13736 | 305 | 66 | 0 | AG | CG |
| 0153 | 10353 | 289 | 70 | 12 | AG | CG |
| 0154 | 12379 | 253 | 80 | 42 | AA | GG |
| 0157 | 14071 | 295 | 44 | 5 | GG | CC |
| 0158 | 11689 | 289 | 47 | 10 | AG | CG |
| 0159 | 12172 | 275 | 40 | 14 | AG | CG |
| 0160 | 13454 | 272 | 96 | 26 | GG | CG |
| 0161 | 13907 | 267 | 45 | 29 | GG | CC |
| 0165 | 09934 | 287 | 48 | 14 | AG | CG |
| 0166 | 12235 | 276 | 68 | 19 | AA | GG |
| 0167 | 11822 | 277 | 35 | 20 | AA | GG |
| 0168 | 12574 | 279 | 51 | 13 | GG | CC |
| 0174 | 13919 | 275 | 76 | 24 | AG | CG |
| 0175 | 14365 | 275 | 61 | 21 | GG | CC |
| 0176 | 11478 | 269 | 48 | 27 | AG | CG |
| 0177 | 12706 | 301 | 73 | 0 | AG | CG |
| 0178 | 14659 | 300 | 36 | 1 | GG | CC |
| 0179 | 13297 | 285 | 74 | 11 | AG | CG |
| 0180 | 14231 | 300 | 40 | 1 | AA | GG |
| 0271 | 14488 | 264 | 110 | 27 | AA | GG |
| 0272 | 13720 | 281 | 47 | 1 | AG | CG |
| 0273 | 12255 | 343 | 65 | 0 | AG | CG |
| 0274 | 11985 | 284 | 43 | 12 | AA | GG |
| 0276 | 09769 | 264 | 47 | 24 | AG | CG |
| 0277 | 15010 | 267 | 85 | 31 | GG | CC |
| 0278 | 15708 | 302 | 40 | 0 | GG | CC |
| 0279 | 11313 | 294 | 64 | 7 | AA | GG |
| 0280 | 15829 | 343 | 33 | 0 | AG | CG |
| 0281 | 15588 | 268 | 116 | 31 | AG | CG |
| 0282 | 12687 | 271 | 104 | 25 | AA | GG |
| 0283 | 10926 | 256 | 50 | 28 | GG | CG |
| 0284 | 15109 | 266 | 51 | 34 | GG | CC |
| 0285 | 13096 | 255 | 128 | 41 | AG | CG |
| 0286 | 10265 | 296 | 83 | 5 | AG | CG |
| 0288 | 14756 | 308 | 39 | 0 | AG | CG |
| 0289 | 15351 | 261 | 87 | 30 | AA | GG |
| 0290 | 14988 | 280 | 108 | 21 | AG | CG |
| 0291 | 12531 | 299 | 80 | 2 | AG | CG |
| 0292 | 12321 | 328 | 54 | 0 | AG | CG |
| 0293 | 14570 | 280 | 76 | 13 | AG | CG |
| 0294 | 11023 | 274 | 86 | 19 | AG | CG |
| 0295 | 14205 | 251 | 146 | 46 | AG | CG |
| 0296 | 14596 | 258 | 101 | 39 | GG | CC |
| 0297 | 13321 | 288 | 68 | 12 | GG | CC |
| 0298 | 10783 | 312 | 40 | 0 | AA | GG |
| 0299 | 12978 | 286 | 53 | 11 | GG | GG |
| 0301 | 15843 | 273 | 85 | 23 | AA | GG |
| 0302 | 13204 | 274 | 90 | 17 | GG | CC |
| 0303 | 14111 | 254 | 76 | 42 | AA | GG |
| 0304 | 14301 | 259 | 84 | 30 | AA | GG |
| 0307 | 12199 | 246 | 105 | 46 | AG | CG |
| 0308 | 15033 | 272 | 117 | 25 | AA | GG |
| 0309 | 10889 | 269 | 52 | 29 | AA | GG |
| 0310 | 12351 | 276 | 39 | 21 | AA | GG |
| 0401 | 15536 | 306 | 92 | 0 | AG | CG |
| 0402 | 13882 | 267 | 53 | 29 | AA | GG |
| 0403 | 12697 | 343 | 46 | 0 | AA | GG |
| 0404 | 12063 | 270 | 102 | 28 | AG | CG |
| 0405 | 10338 | 256 | 70 | 40 | AG | CG |
| 0406 | 10201 | 288 | 84 | 10 | AG | CG |
| 0407 | 11246 | 259 | 58 | 27 | GG | CC |
| 0408 | 10337 | 318 | 45 | 0 | GG | CC |
| 0410 | 14358 | 278 | 70 | 16 | AG | CG |
| 0411 | 15314 | 240 | 89 | 42 | AG | CG |
| 0412 | 12602 | 271 | 51 | 26 | GG | CC |
| 0413 | 10417 | 276 | 71 | 21 | GG | CC |
| 0414 | 10873 | 300 | 67 | 1 | GG | CC |
| 0415 | 10282 | 277 | 102 | 23 | AG | CG |
| 0416 | 12997 | 278 | 78 | 21 | AA | GG |
| 0417 | 13150 | 276 | 52 | 22 | GG | CC |
| 0419 | 14496 | 266 | 79 | 35 | GG | CC |
| 0420 | 10803 | 286 | 115 | 15 | AG | CG |
| 0421 | 09959 | 276 | 116 | 22 | AA | GG |
| 0422 | 15540 | 260 | 45 | 37 | AA | GG |
| 0425 | 15909 | 280 | 91 | 17 | AA | GG |
| 0427 | 10268 | 269 | 74 | 32 | AG | CG |
| 0429 | 10371 | 298 | 57 | 3 | AG | CG |
| 0430 | 11713 | 262 | 86 | 35 | AA | GG |
| 0431 | 12514 | 294 | 48 | 5 | AG | CG |
| 0432 | 13022 | 307 | 86 | 0 | AG | CG |
| 0433 | 15044 | 272 | 55 | 27 | GG | CC |
| 0435 | 14817 | 284 | 89 | 17 | AG | CG |
| 0437 | 14727 | 266 | 129 | 32 | AA | GG |
| 0438 | 12817 | 303 | 53 | 0 | AG | CG |
| 0439 | 13336 | 308 | 42 | 0 | AG | CG |
| 0440 | 10961 | 267 | 92 | 28 | AG | CG |
| 0531 | 13152 | 299 | 102 | 2 | AA | GG |
| 0533 | 12383 | 240 | 98 | 50 | AA | GG |
| 0534 | 14923 | 250 | 64 | 7 | GG | CC |
| 0535 | 15030 | 241 | 86 | 30 | GG | CC |
| 0536 | 10046 | 288 | 33 | 13 | AA | GG |
| 0538 | 13276 | 272 | 64 | 28 | GG | CC |
| 0539 | 11718 | 281 | 48 | 16 | AA | GG |
| 0540 | 14294 | 263 | 104 | 35 | AG | CG |
| 0541 | 14396 | 308 | 42 | 0 | AG | CG |
| 0542 | 12811 | 287 | 69 | 14 | AA | GG |
| 0543 | 12525 | 307 | 31 | 0 | AA | GG |
| 0544 | 14699 | 257 | 105 | 34 | AA | GG |
| 0545 | 15635 | 268 | 83 | 28 | AG | CG |
| 0546 | 14083 | 290 | 59 | 10 | GG | CC |
| 0550 | 13223 | 297 | 60 | 4 | GG | CC |
| 0551 | 10066 | 256 | 65 | 42 | AG | CG |
| 0552 | 15558 | 291 | 94 | 10 | AA | GG |
| 0553 | 13131 | 278 | 66 | 23 | AG | GG |
| 0554 | 10327 | 306 | 44 | 0 | AA | GG |
| 0555 | 15329 | 267 | 65 | 24 | GG | CC |
| 0556 | 15567 | 287 | 59 | 12 | AG | CG |
| 0559 | 14855 | 254 | 81 | 43 | AA | GG |
| 0561 | 11628 | 272 | 61 | 20 | AA | GG |
| 0562 | 14075 | 281 | 45 | 15 | GG | CC |
| 0563 | 13972 | 270 | 116 | 30 | AA | GG |
| 0564 | 13989 | 276 | 59 | 23 | AG | GG |
| 0565 | 12599 | 289 | 41 | 12 | AA | GG |
| 0566 | 11281 | 299 | 41 | 2 | AG | CG |
| 0567 | 14656 | 302 | 32 | 0 | AA | GG |
| 0568 | 10198 | 298 | 100 | 3 | AG | CG |
| 0570 | 12951 | 294 | 33 | 7 | AG | CG |
| 0671 | 14436 | 240 | 50 | 40 | GG | CC |
| 0672 | 11865 | 343 | 57 | 0 | GG | CC |
| 0673 | 15601 | 289 | 64 | 10 | AG | CG |
| 0676 | 15528 | 257 | 122 | 41 | AG | CG |
| 0677 | 11339 | 267 | 127 | 30 | AG | CG |
| 0678 | 11017 | 298 | 57 | 2 | AA | GG |
| 0679 | 15623 | 304 | 36 | 0 | AG | CG |
| 0680 | 13603 | 303 | 72 | 0 | AA | GG |
| 0681 | 10284 | 343 | 35 | 0 | GG | CC |
| 0682 | 15029 | 277 | 55 | 16 | AG | CG |
| 0683 | 13151 | 254 | 94 | 41 | AG | CG |
| 0684 | 14588 | 291 | 99 | 9 | AG | CG |
| 0685 | 11483 | 262 | 104 | 36 | GG | CC |
| 0686 | 15867 | 283 | 110 | 16 | AA | GG |
| 0688 | 11086 | 271 | 101 | 25 | AA | GG |
| 0689 | 13645 | 256 | 116 | 38 | AG | CG |
| 0690 | 10867 | 261 | 77 | 24 | AG | CG |
| 0692 | 11331 | 315 | 66 | 0 | AG | CG |
| 0693 | 13558 | 270 | 129 | 31 | GG | CC |
| 0694 | 12524 | 263 | 68 | 37 | AA | GG |
| 0695 | 09804 | 290 | 100 | 9 | GG | CC |
| 0696 | 15861 | 240 | 126 | 51 | AA | GG |
| 0697 | 14704 | 261 | 100 | 32 | AA | GG |
| 0698 | 13572 | 311 | 51 | 0 | AG | CG |
| 0700 | 10949 | 289 | 86 | 12 | AA | GG |
| 0701 | 11175 | 274 | 88 | 16 | AG | CG |
| 0703 | 13474 | 240 | 59 | 36 | AG | CG |
| 0705 | 13873 | 289 | 53 | 11 | GG | CC |
| 0706 | 10568 | 268 | 65 | 28 | GG | CC |
| 0710 | 11078 | 269 | 64 | 14 | AG | CG |
| 0872 | 13866 | 285 | 67 | 15 | GG | CC |
| 0873 | 14289 | 275 | 101 | 17 | AA | GG |
| 0874 | 14347 | 255 | 126 | 44 | AG | GG |
| 0875 | 12348 | 278 | 82 | 23 | GG | CC |
| 0876 | 15447 | 247 | 83 | 40 | GG | CC |
| 0878 | 14444 | 241 | 77 | 54 | AG | CG |
| 0879 | 11239 | 260 | 76 | 37 | AG | CG |
| 0880 | 15576 | 267 | 76 | 33 | AA | GG |
| 0881 | 10578 | 255 | 101 | 42 | GG | CC |
| 0882 | 15653 | 298 | 54 | 3 | AG | CG |
| 0883 | 15175 | 288 | 83 | 13 | AG | CG |
| 0884 | 14208 | 258 | 111 | 41 | GG | CC |
| 0885 | 11607 | 240 | 111 | 53 | AA | GG |
| 0886 | 14862 | 282 | 65 | 19 | AG | CG |
| 0887 | 15617 | 275 | 103 | 23 | GG | CC |
| 0888 | 14502 | 258 | 105 | 41 | AA | GG |
| 0889 | 11399 | 258 | 65 | 39 | AG | CG |
| 0890 | 14004 | 294 | 75 | 7 | AG | CG |
| 0891 | 14654 | 274 | 65 | 25 | AG | CG |
| 0892 | 11842 | 291 | 55 | 10 | AA | GG |
| 0893 | 09949 | 288 | 70 | 12 | AG | CG |
| 0894 | 13583 | 296 | 52 | 5 | GG | CC |
| 0896 | 11987 | 263 | 88 | 30 | AG | CG |
| 0897 | 11874 | 253 | 82 | 32 | AG | CG |
| 0898 | 15214 | 251 | 97 | 36 | AG | GG |
| 0899 | 11332 | 303 | 76 | 0 | AA | GG |
| 0900 | 12528 | 283 | 44 | 11 | AA | GG |
| 0901 | 10720 | 284 | 65 | 7 | GG | CG |
| 0902 | 14219 | 267 | 82 | 34 | AG | CG |
| 0903 | 15452 | 258 | 128 | 40 | GG | CC |
| 0904 | 15294 | 259 | 59 | 16 | AA | GG |
| 0905 | 12623 | 268 | 50 | 30 | AA | GG |
| 0906 | 13631 | 274 | 70 | 25 | AG | CG |
| 0907 | 13287 | 299 | 63 | 2 | AA | GG |
| 0908 | 13573 | 300 | 57 | 1 | AG | CG |
| 0909 | 12558 | 276 | 80 | 23 | AA | GG |
| 0926 | 12981 | 254 | 58 | 39 | AA | GG |
| 0927 | 14411 | 278 | 102 | 20 | AG | CG |
| 0928 | 15655 | 286 | 60 | 14 | GG | CC |
| 0929 | 12361 | 307 | 32 | 0 | AG | CG |
| 0930 | 10554 | 254 | 133 | 47 | AG | CG |
| 0931 | 11818 | 303 | 47 | 0 | AG | CG |
| 0932 | 10865 | 282 | 75 | 8 | AA | GG |
| 0934 | 10952 | 286 | 85 | 11 | AA | CG |
| 0935 | 13955 | 262 | 86 | 30 | AA | GG |
| 0936 | 15700 | 275 | 74 | 19 | GG | CC |
| 0938 | 10937 | 263 | 83 | 35 | GG | CC |
| 0940 | 15454 | 265 | 47 | 27 | GG | CC |
| 0943 | 10306 | 297 | 50 | 3 | AA | GG |
| 0944 | 12087 | 295 | 76 | 6 | AA | GG |
| 0945 | 11377 | 279 | 98 | 17 | AA | GG |
| 0947 | 12726 | 273 | 56 | 20 | GG | CC |
| 0949 | 12595 | 300 | 49 | 1 | AG | CG |
| 0951 | 13216 | 269 | 102 | 27 | GG | CC |
| 0952 | 12127 | 267 | 81 | 25 | AA | GG |
| 0953 | 14538 | 343 | 42 | 0 | AA | GG |
| 0955 | 11541 | 302 | 87 | 0 | GG | CC |
| 0956 | 14753 | 254 | 134 | 44 | GG | CC |
| 0958 | 12616 | 268 | 133 | 32 | GG | CC |
| 0960 | 11964 | 273 | 52 | 25 | AG | CG |
| 0961 | 15223 | 272 | 69 | 23 | AG | GG |
| 0962 | 14295 | 312 | 54 | 0 | AA | GG |
| 0963 | 09869 | 276 | 59 | 20 | GG | CC |
| 1081 | 10643 | 269 | 120 | 30 | AG | CG |
| 1084 | 14065 | 285 | 81 | 11 | AG | CG |
| 1086 | 10915 | 274 | 48 | 26 | GG | CC |
| 1087 | 10940 | 264 | 108 | 35 | AG | CG |
| 1088 | 11231 | 281 | 78 | 18 | AA | GG |
| 1089 | 14823 | 276 | 78 | 21 | GG | CC |
| 1090 | 14799 | 286 | 58 | 15 | GG | CC |
| 1091 | 09921 | 254 | 82 | 39 | AG | CG |
| 1093 | 10489 | 290 | 60 | 10 | AA | GG |
| 1094 | 13419 | 263 | 76 | 34 | GG | CC |
| 1095 | 09834 | 252 | 133 | 34 | AG | CG |
| 1096 | 15066 | 296 | 50 | 4 | AG | CG |
| 1097 | 12585 | 256 | 94 | 43 | AG | CG |
| 1102 | 13821 | 309 | 38 | 0 | GG | CC |
| 1103 | 11578 | 267 | 75 | 30 | AA | GG |
| 1104 | 14560 | 291 | 77 | 9 | AA | GG |
| 1106 | 12382 | 252 | 65 | 41 | AA | GG |
| 1107 | 09887 | 268 | 101 | 31 | AG | GG |
| 1108 | 13344 | 283 | 64 | 5 | AA | GG |
| 1109 | 15543 | 293 | 80 | 8 | AG | CG |
| 1110 | 15190 | 290 | 62 | 10 | GG | CC |
| 1111 | 11472 | 288 | 54 | 5 | GG | CC |
| 1112 | 11772 | 264 | 69 | 32 | AG | CG |
| 1113 | 10864 | 297 | 40 | 2 | AG | CG |
| 1114 | 10761 | 262 | 115 | 36 | AG | CG |
| 1116 | 13130 | 253 | 95 | 40 | GG | CG |
| 1117 | 15212 | 273 | 120 | 27 | AA | GG |
| 1118 | 13740 | 300 | 42 | 1 | GG | CC |
| 1119 | 11601 | 268 | 77 | 22 | AG | CG |
| 1120 | 15682 | 285 | 94 | 13 | AG | CG |
| 1121 | 10618 | 275 | 83 | 24 | AG | CG |
| 1192 | 15761 | 297 | 36 | 4 | AA | GG |
| 1193 | 14666 | 266 | 72 | 14 | AG | CG |
| 1194 | 14939 | 280 | 60 | 16 | AG | CG |
| 1195 | 09794 | 253 | 113 | 38 | AG | CG |
| 1196 | 14412 | 286 | 77 | 12 | GG | CC |
| 1200 | 13243 | 293 | 53 | 8 | GG | CC |
| 1201 | 10757 | 271 | 67 | 26 | GG | CC |
| 1202 | 14009 | 288 | 84 | 11 | AA | GG |
| 1203 | 14900 | 267 | 88 | 27 | AA | GG |
| 1205 | 09952 | 288 | 103 | 12 | AG | CG |
| 1206 | 15350 | 267 | 46 | 20 | AG | CG |
| 1208 | 14639 | 269 | 53 | 32 | GG | CC |
| 1214 | 12371 | 276 | 71 | 23 | AG | CG |
| 1215 | 11081 | 273 | 107 | 27 | AG | CG |
| 1216 | 13811 | 299 | 55 | 2 | GG | CC |
| 1217 | 11219 | 290 | 35 | 9 | AG | CG |
| 1218 | 12310 | 273 | 34 | 13 | GG | CC |
| 1220 | 15758 | 254 | 82 | 44 | AA | GG |
| 1221 | 14178 | 270 | 89 | 25 | AA | GG |
| 1222 | 10510 | 276 | 88 | 24 | GG | CC |
| 1223 | 12390 | 349 | 32 | 0 | AG | CG |
| 1224 | 12005 | 300 | 33 | 1 | GG | CC |
| 1225 | 11358 | 256 | 62 | 16 | AA | CG |
| 1226 | 14557 | 282 | 79 | 12 | AA | GG |
| 1227 | 13692 | 285 | 107 | 11 | GG | CC |
| 1228 | 15604 | 255 | 96 | 40 | AA | GG |
| 1229 | 12486 | 251 | 105 | 39 | GG | CC |
| 1230 | 10197 | 254 | 92 | 14 | AG | CG |
| 1231 | 14717 | 257 | 68 | 35 | AG | CG |
| 1322 | 12989 | 303 | 75 | 0 | AG | CG |
| 1324 | 13614 | 303 | 41 | 0 | AG | CG |
| 1325 | 14846 | 259 | 102 | 40 | AA | GG |
| 1326 | 13197 | 280 | 76 | 15 | AG | CG |
| 1327 | 11346 | 277 | 90 | 22 | GG | CC |
| 1328 | 13309 | 271 | 95 | 21 | AG | CG |
| 1329 | 12163 | 281 | 57 | 16 | GG | CC |
| 1330 | 14535 | 286 | 71 | 7 | AA | GG |
| 1331 | 09975 | 261 | 56 | 26 | AG | CG |
| 1332 | 11440 | 270 | 70 | 31 | AA | GG |
| 1333 | 11832 | 277 | 122 | 21 | AA | GG |
| 1334 | 14343 | 298 | 51 | 3 | AA | GG |
| 1335 | 11711 | 295 | 55 | 5 | AA | GG |
| 1337 | 12333 | 296 | 67 | 5 | GG | CC |
| 1338 | 12933 | 282 | 64 | 19 | AG | CG |
| 1339 | 15487 | 273 | 102 | 26 | AG | CG |
| 1340 | 13018 | 300 | 36 | 1 | AG | CG |
| 1341 | 13214 | 298 | 41 | 2 | GG | CG |
| 1342 | 13794 | 277 | 50 | 22 | AA | GG |
| 1343 | 11931 | 264 | 51 | 33 | AG | CG |
| 1344 | 13618 | 292 | 105 | 9 | AA | GG |
| 1345 | 15381 | 253 | 76 | 17 | AG | CG |
| 1346 | 12907 | 240 | 101 | 43 | GG | CC |
| 1347 | 10401 | 262 | 44 | 10 | AA | GG |
| 1348 | 13200 | 269 | 81 | 24 | AG | CG |
| 1349 | 12179 | 263 | 76 | 8 | AG | CG |
| 1350 | 12617 | 253 | 126 | 42 | AG | CG |
| 1351 | 11183 | 301 | 37 | 0 | AG | CG |
| 1352 | 13870 | 287 | 104 | 10 | AG | CG |
| 1353 | 15352 | 288 | 64 | 8 | AG | CG |
| 1355 | 15433 | 252 | 57 | 44 | AG | CG |
| 1357 | 13889 | 267 | 86 | 32 | AG | CG |
| 1358 | 13867 | 294 | 62 | 6 | AG | CG |
| 1359 | 14357 | 292 | 50 | 9 | AA | GG |
| 1361 | 11670 | 269 | 123 | 29 | AG | CG |
| 1363 | 11251 | 263 | 42 | 28 | AG | CG |
| 1364 | 12105 | 280 | 80 | 16 | AG | CG |
| 1453 | 09914 | 273 | 67 | 27 | AA | GG |
| 1454 | 12519 | 263 | 133 | 36 | AA | GG |
| 1455 | 10095 | 265 | 120 | 32 | AG | CG |
| 1456 | 13329 | 285 | 49 | 11 | AA | GG |
| 1457 | 11804 | 271 | 78 | 28 | AA | GG |
| 1458 | 13361 | 255 | 77 | 40 | AA | GG |
| 1460 | 12883 | 255 | 111 | 40 | GG | CC |
| 1462 | 10021 | 303 | 32 | 0 | AA | GG |
| 1463 | 13068 | 279 | 58 | 13 | AG | GG |
| 1464 | 13874 | 279 | 70 | 18 | AG | CG |
| 1465 | 15690 | 277 | 68 | 18 | AG | CG |
| 1466 | 15210 | 249 | 114 | 47 | AG | GG |
| 1467 | 15250 | 297 | 89 | 4 | GG | CC |
| 1468 | 11184 | 276 | 79 | 21 | AG | CG |
| 1469 | 15654 | 279 | 92 | 20 | AG | CG |
| 1470 | 11265 | 279 | 66 | 16 | AG | CG |
| 1472 | 10747 | 247 | 93 | 47 | AA | GG |
| 1474 | 11255 | 294 | 40 | 7 | AA | GG |
| 1475 | 15891 | 304 | 38 | 0 | AA | GG |
| 1476 | 10776 | 316 | 81 | 0 | AG | CG |
| 1477 | 11532 | 288 | 100 | 9 | AA | GG |
| 1478 | 15399 | 257 | 76 | 42 | AA | GG |
| 1479 | 12565 | 277 | 89 | 16 | AG | CG |
| 1481 | 12274 | 277 | 59 | 23 | AA | GG |
| 1483 | 11344 | 285 | 59 | 15 | GG | CC |
| 1484 | 15883 | 277 | 67 | 15 | AA | GG |
| 1485 | 10304 | 312 | 58 | 0 | AA | GG |
| 1486 | 12177 | 269 | 98 | 31 | AG | CG |
| 1488 | 13371 | 266 | 80 | 31 | AA | GG |
| 1489 | 14067 | 250 | 60 | 23 | GG | CC |
| 1490 | 13115 | 250 | 110 | 21 | AG | CG |
| 1492 | 14708 | 250 | 139 | 49 | AA | GG |
| 1493 | 11682 | 285 | 54 | 12 | AA | GG |
| 1494 | 12028 | 270 | 82 | 28 | AA | GG |
| 1495 | 12768 | 253 | 120 | 45 | AG | CG |
| 1606 | 17249 | 280 | 37 | 15 | AA | GG |
| 1609 | 16806 | 308 | 74 | 0 | GG | CC |
| 1611 | 21905 | 282 | 82 | 18 | GG | CC |
| 1612 | 18309 | 299 | 88 | 1 | AA | GG |
| 1616 | 19323 | 255 | 88 | 38 | AG | CG |
| 1618 | 21451 | 292 | 57 | 7 | AG | CG |
| 1623 | 18986 | 268 | 45 | 23 | GG | CC |
| 1624 | 19453 | 305 | 30 | 0 | AG | CG |
| 1626 | 16097 | 284 | 43 | 15 | GG | CC |
| 1628 | 21502 | 278 | 59 | 19 | GG | CC |
| 1629 | 17856 | 290 | 40 | 8 | GG | CC |
| 1632 | 21797 | 272 | 76 | 23 | AG | CG |
| 1635 | 16137 | 285 | 54 | 13 | AA | GG |
| 1638 | 17741 | 302 | 36 | 0 | GG | CC |
| 1639 | 19983 | 299 | 65 | 1 | AA | GG |
| 1640 | 19582 | 336 | 56 | 0 | AG | CG |
| 1642 | 15967 | 288 | 88 | 10 | GG | CC |
| 1643 | 20262 | 297 | 76 | 2 | AA | GG |
| 1647 | 21330 | 280 | 30 | 2 | AG | CG |
| 1648 | 20461 | 280 | 58 | 1 | GG | CC |
| 1649 | 20282 | 280 | 70 | 15 | GG | CC |
| 1653 | 16300 | 270 | 60 | 24 | GG | CC |
| 1655 | 18693 | 280 | 44 | 1 | AA | GG |
| 1656 | 21292 | 280 | 79 | 2 | AG | CG |
| 1657 | 20459 | 280 | 30 | 1 | AG | CG |
| 1663 | 18064 | 272 | 92 | 26 | AA | GG |
| 1665 | 19100 | 289 | 43 | 10 | AG | CG |
| 1734 | 21638 | 284 | 92 | 12 | AG | CG |
| 1740 | 17665 | 271 | 45 | 15 | AG | CG |
| 1745 | 18399 | 279 | 110 | 18 | GG | CC |
| 1750 | 16926 | 289 | 84 | 11 | AG | CG |
| 1751 | 17597 | 278 | 49 | 20 | AG | CG |
| 1754 | 21426 | 338 | 40 | 0 | GG | CC |
| 1756 | 19287 | 288 | 36 | 9 | AG | CG |
| 1758 | 16798 | 282 | 67 | 14 | GG | CC |
| 1762 | 21197 | 252 | 80 | 29 | AG | CG |
| 1764 | 20052 | 303 | 54 | 0 | AG | CG |
| 1767 | 17284 | 282 | 52 | 13 | AA | GG |
| 1769 | 18785 | 341 | 33 | 0 | AA | GG |
| 1772 | 21821 | 335 | 36 | 0 | AG | CG |
| 1775 | 16938 | 258 | 40 | 32 | AG | CG |
| 1779 | 17866 | 282 | 80 | 18 | AG | CG |
| 1780 | 17380 | 287 | 39 | 13 | GG | CC |
| 1781 | 19873 | 280 | 38 | 16 | AG | CG |
| 1782 | 18260 | 285 | 78 | 14 | AA | GG |
| 1787 | 20910 | 270 | 37 | 26 | GG | CC |
| 1790 | 21909 | 288 | 91 | 12 | AG | CG |
| 1791 | 20253 | 320 | 59 | 0 | AA | GG |
| 1792 | 18752 | 292 | 68 | 8 | GG | CC |
| 1794 | 16913 | 301 | 66 | 0 | AG | CG |
| 1797 | 19571 | 286 | 47 | 14 | AA | GG |
| 1801 | 17736 | 252 | 115 | 45 | AG | CG |
| 1802 | 20540 | 258 | 84 | 35 | AG | CG |
| 1804 | 17447 | 269 | 78 | 30 | AG | CG |
| 1809 | 17833 | 252 | 84 | 48 | GG | CC |
| 1812 | 20147 | 270 | 108 | 26 | AG | CG |
| 1813 | 21783 | 270 | 91 | 28 | GG | CC |
| 1818 | 22047 | 253 | 132 | 42 | GG | CC |
| 1820 | 16689 | 268 | 66 | 27 | AA | GG |
| 1822 | 20896 | 271 | 104 | 29 | AA | GG |
| 1825 | 19591 | 263 | 77 | 36 | AG | CG |
| 1827 | 18257 | 261 | 89 | 34 | AG | CG |
| 1828 | 20352 | 271 | 86 | 28 | AG | CG |
| 1832 | 21552 | 248 | 80 | 49 | AA | GG |
| 1835 | 17602 | 278 | 61 | 22 | AA | GG |
| 1838 | 22002 | 273 | 102 | 26 | AG | CG |
| 1839 | 20733 | 268 | 126 | 30 | GG | CC |
| 1840 | 20708 | 248 | 111 | 50 | GG | CC |
| 1844 | 19669 | 259 | 106 | 37 | AG | CG |
| 1845 | 18908 | 267 | 67 | 30 | AA | GG |
| 1847 | 21356 | 271 | 109 | 29 | AA | GG |
| 1849 | 20829 | 262 | 62 | 37 | AG | CG |
| 1852 | 18980 | 267 | 77 | 31 | AG | CG |
| 1854 | 18593 | 272 | 71 | 27 | AG | CG |
| 1855 | 19498 | 261 | 76 | 33 | AG | CG |
| 1857 | 19529 | 254 | 115 | 43 | AG | CG |
| 1859 | 19482 | 267 | 73 | 29 | GG | CC |
| 1861 | 20947 | 249 | 115 | 46 | AG | CG |
| 1862 | 17429 | 243 | 116 | 57 | AA | GG |
| 1863 | 21611 | 258 | 96 | 37 | AG | CG |
| 1873 | 20751 | 246 | 124 | 53 | AG | CG |
| 1874 | 19296 | 257 | 63 | 39 | AG | CG |
| 1875 | 19457 | 272 | 49 | 10 | AG | CG |
| 1876 | 21105 | 267 | 78 | 30 | AA | GG |
| 1877 | 19122 | 264 | 38 | 33 | AG | CG |
| 1878 | 21179 | 267 | 60 | 29 | AG | CG |
| 1879 | 20445 | 278 | 39 | 1 | AG | CG |
| 1880 | 20038 | 259 | 40 | 32 | GG | CC |
| 1881 | 20310 | 288 | 44 | 7 | GG | CC |
| 1882 | 18905 | 270 | 95 | 26 | AA | GG |
| 1883 | 16994 | 279 | 63 | 20 | AG | CG |
| 1884 | 17658 | 255 | 105 | 43 | AA | GG |
| 1885 | 19750 | 280 | 41 | 16 | GG | CC |
| 1886 | 20690 | 277 | 35 | 18 | GG | CC |
| 1887 | 19031 | 309 | 30 | 0 | AG | CG |
| 1896 | 16679 | 283 | 64 | 14 | AA | GG |
| 1899 | 19531 | 272 | 33 | 26 | AG | CG |
| 1900 | 19685 | 272 | 70 | 22 | AG | CG |
| 1901 | 18405 | 272 | 84 | 24 | AG | CG |
| 1904 | 16421 | 298 | 52 | 2 | GG | CC |
| 1905 | 15982 | 267 | 50 | 18 | AG | CG |
| 1906 | 19935 | 272 | 69 | 28 | AA | GG |
| 1914 | 18480 | 290 | 80 | 10 | AA | GG |
| 1915 | 18711 | 260 | 40 | 27 | AG | CG |
| 1921 | 18115 | 294 | 44 | 6 | AG | CG |
| 1922 | 20381 | 298 | 31 | 2 | AA | GG |
| 1925 | 16702 | 295 | 45 | 3 | AG | CG |
| 1929 | 17637 | 278 | 71 | 21 | AG | CG |
| 2007 | 21038 | 286 | 68 | 12 | AG | CG |
| 2011 | 17812 | 279 | 85 | 18 | GG | CG |
| 2012 | 18859 | 275 | 44 | 20 | AG | CG |
| 2013 | 20258 | 288 | 72 | 11 | AG | CG |
| 2015 | 17711 | 278 | 86 | 19 | AA | GG |
| 2016 | 19021 | 285 | 74 | 13 | AG | CG |
| 2017 | 19054 | 271 | 46 | 26 | AA | GG |
| 2018 | 18690 | 263 | 37 | 31 | GG | CC |
| 2020 | 18573 | 280 | 69 | 16 | GG | CC |
| 2021 | 19445 | 284 | 36 | 13 | AG | CG |
| 2024 | 20313 | 288 | 45 | 9 | GG | CC |
| 2025 | 21506 | 280 | 31 | 15 | AG | CG |
| 2027 | 18692 | 291 | 32 | 9 | AG | CG |
| 2028 | 21713 | 312 | 31 | 0 | AA | GG |
| 2029 | 17016 | 274 | 39 | 24 | AG | CG |
| 2031 | 19742 | 270 | 72 | 30 | GG | CC |
| 2033 | 20922 | 276 | 56 | 17 | AG | CG |
| 2036 | 20279 | 283 | 93 | 15 | AG | CG |
| 2040 | 19553 | 303 | 50 | 0 | AA | GG |
| 2044 | 16299 | 289 | 36 | 9 | GG | CC |
| 2045 | 16890 | 269 | 78 | 25 | AG | CG |
| 2048 | 19414 | 269 | 38 | 23 | AA | GG |
| 2049 | 20421 | 293 | 50 | 7 | GG | CC |
| 2053 | 17709 | 256 | 45 | 1 | AA | GG |
| 2054 | 19049 | 286 | 34 | 14 | AA | GG |
| 2058 | 19863 | 263 | 92 | 37 | GG | CC |
| 2061 | 20003 | 316 | 50 | 0 | AA | GG |
| 2063 | 19071 | 297 | 65 | 3 | AG | CG |
| 2064 | 17166 | 285 | 50 | 12 | AA | GG |
| 2067 | 17565 | 280 | 81 | 20 | AG | CG |
| 2141 | 19611 | 241 | 40 | 40 | GG | CC |
| 2143 | 20102 | 275 | 97 | 17 | AG | CG |
| 2145 | 16094 | 275 | 53 | 22 | GG | CC |
| 2146 | 20864 | 275 | 80 | 19 | AG | CG |
| 2152 | 19334 | 281 | 69 | 19 | GG | CC |
| 2156 | 20181 | 301 | 70 | 0 | AA | GG |
| 2158 | 19501 | 277 | 42 | 22 | AG | CG |
| 2159 | 16493 | 301 | 41 | 0 | GG | CC |
| 2160 | 18953 | 285 | 44 | 15 | AG | CG |
| 2164 | 20604 | 271 | 47 | 29 | AG | CG |
| 2169 | 17620 | 268 | 33 | 32 | AA | GG |
| 2172 | 19890 | 305 | 57 | 0 | GG | CC |
| 2174 | 19794 | 298 | 59 | 2 | AG | CG |
| 2176 | 17807 | 336 | 35 | 0 | GG | CG |
| 2178 | 16117 | 317 | 44 | 0 | AG | CG |
| 2183 | 21236 | 283 | 56 | 15 | AG | CG |
| 2186 | 21262 | 259 | 56 | 30 | AA | GG |
| 2308 | 17592 | 237 | 153 | 62 | AA | GG |
| 2309 | 21839 | 270 | 68 | 27 | AG | CG |
| 2311 | 17735 | 263 | 102 | 34 | GG | CC |
| 2312 | 21582 | 244 | 96 | 53 | GG | CC |
| 2314 | 21224 | 270 | 111 | 27 | GG | CC |
| 2321 | 19365 | 247 | 105 | 51 | AG | CG |
| 2323 | 21138 | 258 | 53 | 35 | AA | GG |
| 2328 | 19811 | 264 | 111 | 30 | AG | CG |
| 2330 | 20854 | 262 | 135 | 35 | AA | GG |
| 2332 | 20239 | 274 | 81 | 21 | AA | GG |
| 2333 | 21563 | 258 | 58 | 40 | GG | CC |
| 2334 | 17010 | 278 | 33 | 14 | GG | CC |
| 2335 | 20901 | 276 | 51 | 15 | AG | CG |
| 2336 | 19437 | 293 | 69 | 6 | GG | CC |
| 2337 | 18782 | 301 | 84 | 0 | AA | GG |
| 2338 | 16783 | 297 | 38 | 2 | GG | CC |
| 2339 | 20014 | 270 | 111 | 28 | AG | CG |
| 2340 | 20456 | 269 | 89 | 30 | AG | CG |
| 2341 | 19080 | 258 | 82 | 32 | AG | CG |
| 2342 | 18320 | 284 | 44 | 13 | AG | CG |
| 2344 | 19275 | 271 | 39 | 19 | AA | GG |
| 2345 | 17677 | 279 | 78 | 21 | AA | GG |
| 2346 | 16805 | 280 | 108 | 16 | GG | CC |
| 2347 | 19855 | 265 | 65 | 30 | GG | CC |
| 2349 | 21617 | 291 | 67 | 9 | AA | GG |
| 2352 | 19972 | 278 | 38 | 17 | AG | CG |
| 2353 | 17087 | 335 | 51 | 0 | AA | GG |
| 2355 | 19084 | 268 | 67 | 20 | AG | CG |
| 2356 | 18120 | 335 | 38 | 0 | GG | CC |
| 2357 | 20032 | 292 | 77 | 8 | GG | CG |
| 2358 | 21653 | 239 | 80 | 47 | GG | CC |
| 2359 | 21001 | 270 | 104 | 25 | AG | CG |
| 2360 | 20828 | 282 | 103 | 19 | AG | CG |
| 2361 | 16125 | 269 | 60 | 29 | AA | GG |
| 2363 | 21171 | 249 | 132 | 51 | GG | CC |
| 2364 | 18087 | 283 | 72 | 10 | AG | CG |
| 2365 | 20093 | 258 | 43 | 24 | AA | GG |
| 2366 | 17855 | 286 | 52 | 11 | GG | CC |
| 2368 | 19541 | 317 | 69 | 0 | AA | GG |
| 2369 | 21483 | 265 | 34 | 23 | AA | GG |
| 2370 | 19783 | 256 | 135 | 42 | AA | GG |
| 2371 | 20716 | 243 | 120 | 55 | GG | CC |
| 2372 | 16487 | 245 | 148 | 49 | AG | CG |
| 2373 | 21850 | 261 | 121 | 31 | GG | CC |
| 2375 | 17285 | 257 | 89 | 32 | AA | GG |
| 2377 | 20946 | 248 | 120 | 48 | AA | GG |
| 2379 | 20717 | 259 | 91 | 35 | AG | CG |
| 2380 | 22042 | 262 | 99 | 32 | GG | CC |
| 2382 | 18360 | 278 | 52 | 21 | GG | CC |
| 2383 | 21701 | 258 | 90 | 38 | AG | GG |
| 2385 | 20440 | 257 | 132 | 42 | AG | CG |
| 2386 | 21556 | 257 | 65 | 34 | AA | GG |
| 2387 | 17194 | 267 | 74 | 23 | AG | CG |
| 2388 | 21333 | 249 | 90 | 48 | AG | CG |
| 2389 | 19709 | 290 | 64 | 9 | GG | CC |
| 2390 | 17217 | 274 | 68 | 10 | AA | GG |
| 2391 | 19356 | 254 | 137 | 41 | GG | CG |
| 2392 | 20815 | 276 | 62 | 17 | AG | CG |
| 2394 | 21733 | 244 | 144 | 55 | GG | CC |
| 2395 | 18406 | 253 | 138 | 43 | AG | CG |
| 2401 | 18734 | 265 | 90 | 29 | AG | CG |
| 2402 | 17864 | 286 | 62 | 13 | AA | GG |
| 2403 | 16460 | 268 | 96 | 31 | AA | GG |
| 2404 | 16991 | 285 | 98 | 11 | AG | CG |
| 2406 | 17698 | 269 | 120 | 27 | AA | GG |
| 2407 | 16131 | 294 | 42 | 5 | AA | GG |
| 2408 | 18397 | 262 | 66 | 32 | AG | CG |
| 2409 | 16816 | 267 | 33 | 33 | GG | CC |
| 2410 | 18539 | 267 | 88 | 32 | AA | GG |
| 2411 | 16511 | 320 | 46 | 0 | AG | CG |
| 2412 | 20957 | 285 | 94 | 11 | AA | GG |
| 2413 | 18900 | 288 | 50 | 9 | AG | CG |
| 2414 | 19874 | 288 | 36 | 10 | AA | GG |
| 2419 | 20467 | 290 | 76 | 10 | AG | CG |
| 2426 | 21360 | 261 | 37 | 31 | AA | GG |
| 2427 | 18956 | 304 | 45 | 0 | GG | CC |
| 2431 | 16973 | 285 | 63 | 14 | AG | CG |
| 2433 | 21418 | 287 | 45 | 11 | AG | CG |
| 2435 | 16947 | 336 | 41 | 0 | AG | CG |
| 2440 | 19826 | 287 | 86 | 13 | AG | CG |
| 2443 | 19751 | 283 | 33 | 15 | GG | CC |
| 2445 | 20247 | 288 | 107 | 10 | AA | GG |
| 2446 | 17057 | 284 | 74 | 13 | AA | GG |
| 2451 | 18983 | 290 | 66 | 10 | GG | CC |
| 2452 | 20995 | 255 | 54 | 45 | AG | CG |
| 2454 | 19108 | 284 | 65 | 15 | AG | CG |
| 2458 | 18334 | 257 | 31 | 31 | AG | CG |
| 2459 | 16533 | 335 | 61 | 0 | AA | GG |
| 2465 | 17743 | 266 | 36 | 19 | AG | CG |
| 2468 | 17165 | 291 | 65 | 9 | AG | CG |
| 2470 | 16504 | 296 | 42 | 4 | AG | CG |
| 2529 | 19483 | 294 | 39 | 5 | GG | CC |
| 2533 | 20625 | 309 | 77 | 0 | AG | CG |
| 2538 | 18653 | 262 | 66 | 30 | AG | CG |
| 2542 | 16641 | 278 | 76 | 19 | AG | CG |
| 2543 | 19029 | 302 | 34 | 0 | GG | CC |
| 2547 | 20951 | 251 | 88 | 38 | AG | CG |
| 2552 | 19530 | 251 | 41 | 34 | AG | CG |
| 2556 | 22113 | 270 | 80 | 25 | AA | GG |
| 2557 | 18127 | 285 | 59 | 15 | GG | CC |
| 2560 | 20105 | 295 | 47 | 4 | AG | CG |
| 2562 | 21471 | 291 | 79 | 9 | GG | CG |
| 2563 | 18103 | 285 | 46 | 14 | AG | CG |
| 2566 | 16655 | 288 | 36 | 11 | AG | CG |
| 2567 | 19315 | 268 | 71 | 23 | AA | GG |
| 2569 | 21379 | 282 | 38 | 17 | AG | CG |
| 2572 | 20280 | 284 | 50 | 13 | GG | CC |
| 2574 | 18374 | 270 | 41 | 30 | AA | GG |
| 2575 | 19903 | 291 | 46 | 9 | AG | CG |
| 2576 | 20945 | 286 | 48 | 13 | GG | CC |
| 2577 | 18756 | 291 | 50 | 8 | AG | CG |
| 2586 | 21676 | 251 | 62 | 38 | GG | CC |
| 2895 | 21097 | 249 | 65 | 38 | GG | CC |
| 2896 | 17728 | 251 | 100 | 39 | AA | GG |
| 2902 | 21482 | 267 | 86 | 31 | AG | CG |
| 2906 | 20402 | 273 | 98 | 22 | AA | GG |
| 2907 | 21414 | 247 | 97 | 45 | AG | CG |
| 2910 | 21101 | 249 | 149 | 50 | AG | CG |
| 2912 | 20221 | 256 | 72 | 36 | GG | CC |
| 2915 | 18789 | 250 | 64 | 41 | GG | CC |
| 2918 | 21890 | 239 | 95 | 45 | AG | CG |
| 2919 | 17389 | 252 | 131 | 46 | AA | GG |
| 2920 | 20083 | 267 | 78 | 32 | AA | GG |
| 2921 | 15968 | 250 | 123 | 44 | AA | GG |
| 2923 | 20135 | 254 | 116 | 45 | AA | GG |
| 2937 | 17696 | 264 | 108 | 33 | AG | CG |
| 2950 | 18748 | 264 | 113 | 36 | GG | CC |
| 2951 | 19019 | 257 | 90 | 32 | AG | CG |
| 2954 | 20399 | 263 | 67 | 31 | AA | GG |
| 2958 | 19422 | 261 | 119 | 35 | AA | GG |
| 2959 | 20263 | 270 | 121 | 29 | AG | CG |
| 2961 | 17334 | 251 | 98 | 40 | GG | CC |
| 2962 | 19884 | 246 | 143 | 49 | AG | CG |
| 2964 | 17802 | 258 | 97 | 27 | AA | GG |
| 2965 | 17834 | 272 | 112 | 28 | AA | GG |
| 2967 | 22098 | 258 | 137 | 41 | AG | CG |
| 2969 | 16400 | 257 | 110 | 41 | AA | GG |
| 2974 | 20822 | 266 | 101 | 27 | AG | CG |
| 2983 | 21460 | 251 | 97 | 47 | AA | GG |
| 2985 | 20148 | 255 | 82 | 31 | GG | CC |
| 2988 | 17435 | 255 | 106 | 38 | AG | CG |
| 2991 | 17857 | 249 | 83 | 41 | AG | CG |
| 3050 | 18626 | 255 | 108 | 44 | AG | CG |
| 3051 | 18788 | 245 | 105 | 52 | AG | GG |
| 3054 | 20693 | 247 | 86 | 49 | AG | GG |
| 3055 | 20750 | 243 | 138 | 57 | AG | CG |
| 3057 | 20137 | 244 | 97 | 54 | AG | CG |
| 3058 | 19697 | 258 | 86 | 40 | AG | CG |
| 3059 | 16709 | 273 | 102 | 25 | AA | GG |
| 3062 | 21606 | 245 | 75 | 28 | GG | CC |
| 3063 | 19587 | 245 | 80 | 35 | AG | CG |
| 3065 | 20712 | 242 | 136 | 53 | AG | CG |
| 3067 | 22048 | 237 | 125 | 58 | AG | CG |
| 3068 | 20574 | 266 | 61 | 25 | AA | GG |
| 3073 | 19304 | 255 | 75 | 40 | AA | GG |
| 3074 | 17438 | 266 | 67 | 30 | GG | CC |
| 3075 | 16891 | 248 | 96 | 48 | GG | CC |
| 3083 | 15966 | 254 | 129 | 44 | AA | GG |
| 3084 | 16291 | 249 | 80 | 45 | AG | CG |
| 3085 | 17700 | 269 | 96 | 31 | AG | CG |
| 3089 | 20454 | 268 | 84 | 31 | AA | GG |
| 3092 | 21226 | 260 | 61 | 40 | GG | CG |
| 3093 | 17801 | 270 | 99 | 24 | AA | GG |
| 3094 | 19485 | 246 | 112 | 55 | AG | CG |
| 3096 | 17730 | 252 | 103 | 47 | AG | CG |
| 3099 | 21230 | 249 | 107 | 46 | AG | CG |
| 3109 | 20967 | 300 | 126 | 0 | AG | CG |
| 3110 | 20934 | 252 | 105 | 41 | AA | GG |
| 3111 | 20900 | 263 | 68 | 31 | AG | CG |
| 3113 | 16849 | 260 | 99 | 34 | AA | GG |
| 3117 | 20325 | 267 | 92 | 29 | GG | CG |
| 3118 | 21554 | 248 | 137 | 51 | AG | CG |
| 3121 | 20774 | 256 | 75 | 44 | AG | GG |

**Notes.**

CAGE=cage number; cx_wb=wing number; FEA = first egg age; E59W = egg number at age 59 weeks; E300D = egg number at age 300 days.

**Supplementary table S3 Raw data of association at DRD2 gene**

| CAGE | cx_wb | FEA | E59W | E300D | C+7T | C+364G |
| --- | --- | --- | --- | --- | --- | --- |
| 0022 | 11280 | 290 | 38 | 8 | CC | CG |
| 0023 | 13211 | 294 | 72 | 10 | TT | GG |
| 0024 | 13734 | 275 | 60 | 22 | CC | GG |
| 0025 | 13954 | 262 | 69 | 25 | CT | CG |
| 0026 | 12173 | 276 | 55 | 20 | CC | GG |
| 0027 | 13487 | 281 | 45 | 18 | TT | CC |
| 0028 | 13491 | 290 | 63 | 11 | CT | CG |
| 0030 | 13599 | 276 | 57 | 20 | CT | CG |
| 0031 | 12953 | 240 | 150 | 49 | CC | GG |
| 0033 | 14285 | 296 | 45 | 5 | TT | GG |
| 0034 | 11792 | 255 | 111 | 47 | CT | CG |
| 0035 | 14561 | 298 | 90 | 2 | CT | CG |
| 0036 | 10100 | 298 | 68 | 3 | CC | GG |
| 0037 | 15511 | 275 | 80 | 24 | CC | GG |
| 0038 | 14207 | 292 | 68 | 8 | CT | CG |
| 0039 | 15512 | 259 | 74 | 37 | CC | GG |
| 0044 | 15062 | 280 | 54 | 22 | CT | CG |
| 0045 | 15469 | 265 | 103 | 29 | CT | CG |
| 0046 | 15233 | 310 | 58 | 0 | CC | GG |
| 0048 | 12288 | 255 | 78 | 33 | CC | GG |
| 0049 | 11054 | 289 | 73 | 12 | CC | GG |
| 0050 | 11685 | 270 | 106 | 26 | CC | GG |
| 0051 | 15306 | 284 | 72 | 17 | CC | GG |
| 0052 | 14405 | 295 | 60 | 4 | CT | CG |
| 0053 | 13186 | 290 | 89 | 9 | CC | GG |
| 0054 | 09840 | 274 | 107 | 27 | CC | GG |
| 0056 | 13063 | 293 | 66 | 8 | CC | GG |
| 0057 | 15392 | 289 | 44 | 12 | CC | GG |
| 0059 | 15068 | 287 | 46 | 13 | CT | CG |
| 0060 | 14633 | 293 | 65 | 8 | CC | GG |
| 0141 | 13743 | 288 | 47 | 9 | CC | GG |
| 0142 | 13674 | 289 | 107 | 12 | CT | CG |
| 0143 | 10870 | 310 | 37 | 0 | CT | GG |
| 0145 | 10254 | 293 | 33 | 6 | CT | GG |
| 0146 | 14985 | 262 | 139 | 37 | CC | CG |
| 0147 | 12395 | 282 | 43 | 18 | CC | GG |
| 0148 | 12085 | 267 | 83 | 31 | CT | CG |
| 0149 | 13687 | 279 | 70 | 22 | CC | GG |
| 0150 | 15596 | 289 | 48 | 5 | TT | CC |
| 0151 | 14867 | 256 | 95 | 43 | CC | GG |
| 0152 | 13736 | 305 | 66 | 0 | CC | GG |
| 0153 | 10353 | 289 | 70 | 12 | CC | GG |
| 0154 | 12379 | 253 | 80 | 42 | CT | CG |
| 0157 | 14071 | 295 | 44 | 5 | CT | CG |
| 0158 | 11689 | 289 | 47 | 10 | CT | CG |
| 0159 | 12172 | 275 | 40 | 14 | CC | GG |
| 0160 | 13454 | 272 | 96 | 26 | CT | CG |
| 0161 | 13907 | 267 | 45 | 29 | CC | GG |
| 0164 | 11466 | 279 | 66 | 22 | CC | GG |
| 0165 | 09934 | 287 | 48 | 14 | CC | GG |
| 0167 | 11822 | 277 | 35 | 20 | CC | GG |
| 0168 | 12574 | 279 | 51 | 13 | CC | GG |
| 0176 | 11478 | 269 | 48 | 27 | CC | GG |
| 0178 | 14659 | 300 | 36 | 1 | CT | CG |
| 0271 | 14488 | 264 | 110 | 27 | CC | GG |
| 0272 | 13720 | 281 | 47 | 1 | CC | GG |
| 0274 | 11985 | 284 | 43 | 12 | CC | GG |
| 0276 | 09769 | 264 | 47 | 24 | CC | GG |
| 0278 | 15708 | 302 | 40 | 0 | CT | CG |
| 0279 | 11313 | 294 | 64 | 7 | TT | GG |
| 0281 | 15588 | 268 | 116 | 31 | CT | CG |
| 0282 | 12687 | 271 | 104 | 25 | CC | GG |
| 0285 | 13096 | 255 | 128 | 41 | CT | CG |
| 0286 | 10265 | 296 | 83 | 5 | CC | GG |
| 0289 | 15351 | 261 | 87 | 30 | CT | CG |
| 0290 | 14988 | 280 | 108 | 21 | CT | CG |
| 0291 | 12531 | 299 | 80 | 2 | CT | CG |
| 0292 | 12321 | 328 | 54 | 0 | CC | GG |
| 0293 | 14570 | 280 | 76 | 13 | CT | CG |
| 0294 | 11023 | 274 | 86 | 19 | CC | GG |
| 0295 | 14205 | 251 | 146 | 46 | CT | CG |
| 0296 | 14596 | 258 | 101 | 39 | CC | GG |
| 0297 | 13321 | 288 | 68 | 12 | CT | CG |
| 0298 | 10783 | 312 | 40 | 0 | CC | GG |
| 0299 | 12978 | 286 | 53 | 11 | CT | CG |
| 0301 | 15843 | 273 | 85 | 23 | CC | GG |
| 0302 | 13204 | 274 | 90 | 17 | CT | CG |
| 0303 | 14111 | 254 | 76 | 42 | CT | CG |
| 0308 | 15033 | 272 | 117 | 25 | CC | GG |
| 0309 | 10889 | 269 | 52 | 29 | CT | CG |
| 0402 | 13882 | 267 | 53 | 29 | CC | GG |
| 0404 | 12063 | 270 | 102 | 28 | CT | CG |
| 0405 | 10338 | 256 | 70 | 40 | CC | GG |
| 0406 | 10201 | 288 | 84 | 10 | CC | GG |
| 0408 | 10337 | 318 | 45 | 0 | CT | CG |
| 0410 | 14358 | 278 | 70 | 16 | CC | GG |
| 0411 | 15314 | 240 | 89 | 42 | CT | CG |
| 0415 | 10282 | 277 | 102 | 23 | CC | GG |
| 0416 | 12997 | 278 | 78 | 21 | CC | GG |
| 0417 | 13150 | 276 | 52 | 22 | CC | GG |
| 0421 | 09959 | 276 | 116 | 22 | CT | CG |
| 0422 | 15540 | 260 | 45 | 37 | CT | CG |
| 0423 | 11136 | 305 | 70 | 0 | CT | CG |
| 0425 | 15909 | 280 | 91 | 17 | CC | GG |
| 0427 | 10268 | 269 | 74 | 32 | CC | GG |
| 0429 | 10371 | 298 | 57 | 3 | CT | CG |
| 0430 | 11713 | 262 | 86 | 35 | CC | GG |
| 0431 | 12514 | 294 | 48 | 5 | CT | CG |
| 0432 | 13022 | 307 | 86 | 0 | CC | GG |
| 0433 | 15044 | 272 | 55 | 27 | CT | CG |
| 0435 | 14817 | 284 | 89 | 17 | CC | GG |
| 0437 | 14727 | 266 | 129 | 32 | CT | CG |
| 0438 | 12817 | 303 | 78 | 3 | TT | CC |
| 0439 | 13336 | 308 | 42 | 0 | CT | CG |
| 0440 | 10961 | 267 | 92 | 28 | CC | GG |
| 0531 | 13152 | 299 | 102 | 2 | CC | GG |
| 0533 | 12383 | 240 | 98 | 50 | CC | GG |
| 0534 | 14923 | 250 | 64 | 7 | CT | CG |
| 0535 | 15030 | 241 | 86 | 30 | CC | GG |
| 0536 | 10046 | 288 | 33 | 13 | CT | CG |
| 0538 | 13276 | 272 | 64 | 28 | CC | GG |
| 0539 | 11718 | 281 | 48 | 16 | CC | GG |
| 0540 | 14294 | 263 | 104 | 35 | CC | GG |
| 0542 | 12811 | 287 | 69 | 14 | CC | GG |
| 0544 | 14699 | 257 | 105 | 34 | CT | CG |
| 0545 | 15635 | 268 | 83 | 28 | CC | GG |
| 0546 | 14083 | 290 | 59 | 10 | CC | GG |
| 0548 | 11893 | 277 | 91 | 17 | CC | GG |
| 0550 | 13223 | 297 | 60 | 4 | CT | CG |
| 0551 | 10066 | 256 | 65 | 42 | CC | GG |
| 0552 | 15558 | 291 | 94 | 10 | CC | GG |
| 0553 | 13131 | 278 | 66 | 23 | CT | CG |
| 0554 | 10327 | 306 | 44 | 0 | CT | CG |
| 0555 | 15329 | 267 | 65 | 24 | CC | GG |
| 0556 | 15567 | 287 | 59 | 12 | CT | CG |
| 0559 | 14855 | 254 | 81 | 43 | CC | GG |
| 0561 | 11628 | 272 | 61 | 20 | CC | GG |
| 0562 | 14075 | 281 | 45 | 15 | CC | GG |
| 0563 | 13972 | 270 | 116 | 30 | CC | GG |
| 0564 | 13989 | 276 | 59 | 23 | CC | GG |
| 0565 | 12599 | 289 | 41 | 12 | CC | GG |
| 0566 | 11281 | 299 | 41 | 2 | CT | CG |
| 0567 | 14656 | 302 | 32 | 0 | CT | GG |
| 0568 | 10198 | 298 | 100 | 3 | CC | GG |
| 0570 | 12951 | 294 | 33 | 7 | CC | GG |
| 0671 | 14436 | 240 | 50 | 40 | CC | GG |
| 0672 | 11865 | 343 | 57 | 0 | CT | CG |
| 0673 | 15601 | 289 | 64 | 10 | CT | CG |
| 0676 | 15528 | 257 | 122 | 41 | CC | GG |
| 0677 | 11339 | 267 | 127 | 30 | CC | GG |
| 0678 | 11017 | 298 | 57 | 2 | CC | GG |
| 0679 | 15623 | 304 | 36 | 0 | CC | GG |
| 0680 | 13603 | 303 | 72 | 0 | CC | GG |
| 0681 | 10284 | 343 | 35 | 0 | CT | CG |
| 0682 | 15029 | 277 | 55 | 16 | CT | GG |
| 0683 | 13151 | 254 | 94 | 41 | CC | GG |
| 0684 | 14588 | 291 | 99 | 9 | CC | GG |
| 0685 | 11483 | 262 | 104 | 36 | CT | CG |
| 0686 | 15867 | 283 | 110 | 16 | CT | CG |
| 0688 | 11086 | 271 | 101 | 25 | CC | GG |
| 0689 | 13645 | 256 | 116 | 38 | CT | CG |
| 0690 | 10867 | 261 | 77 | 24 | CT | CG |
| 0693 | 13558 | 270 | 129 | 31 | CT | CG |
| 0694 | 12524 | 263 | 68 | 37 | CT | CG |
| 0696 | 15861 | 240 | 126 | 51 | CC | GG |
| 0697 | 14704 | 261 | 100 | 32 | CC | GG |
| 0698 | 13572 | 311 | 51 | 3 | TT | CC |
| 0700 | 10949 | 289 | 86 | 12 | CC | GG |
| 0701 | 11175 | 274 | 88 | 16 | CC | GG |
| 0705 | 13873 | 289 | 53 | 11 | CC | GG |
| 0706 | 10568 | 268 | 65 | 28 | CC | GG |
| 0710 | 11078 | 269 | 64 | 14 | CC | GG |
| 0872 | 13866 | 285 | 67 | 15 | CT | CG |
| 0873 | 14289 | 275 | 101 | 17 | CC | GG |
| 0874 | 14347 | 255 | 126 | 44 | CC | GG |
| 0875 | 12348 | 278 | 82 | 23 | CT | CG |
| 0876 | 15447 | 247 | 83 | 40 | CC | GG |
| 0878 | 14444 | 241 | 77 | 54 | CC | GG |
| 0879 | 11239 | 260 | 76 | 37 | CT | CG |
| 0880 | 15576 | 267 | 76 | 33 | CC | GG |
| 0881 | 10578 | 255 | 101 | 42 | CC | GG |
| 0882 | 15653 | 298 | 54 | 3 | CC | GG |
| 0884 | 14208 | 258 | 111 | 41 | CT | CG |
| 0885 | 11607 | 240 | 111 | 53 | CC | GG |
| 0886 | 14862 | 282 | 65 | 19 | CC | GG |
| 0887 | 15617 | 275 | 103 | 23 | CT | CG |
| 0888 | 14502 | 258 | 105 | 41 | CT | CG |
| 0889 | 11399 | 258 | 65 | 39 | CC | CG |
| 0890 | 14004 | 294 | 75 | 7 | CC | GG |
| 0891 | 14654 | 274 | 65 | 25 | CT | CG |
| 0892 | 11842 | 291 | 55 | 10 | CC | GG |
| 0893 | 09949 | 288 | 70 | 12 | CT | CG |
| 0894 | 13583 | 296 | 52 | 5 | CC | GG |
| 0896 | 11987 | 263 | 88 | 30 | CC | GG |
| 0897 | 11874 | 253 | 82 | 32 | CC | GG |
| 0898 | 15214 | 251 | 97 | 36 | CC | GG |
| 0900 | 12528 | 283 | 44 | 11 | CC | GG |
| 0901 | 10720 | 284 | 65 | 7 | CC | GG |
| 0902 | 14219 | 267 | 82 | 34 | CC | GG |
| 0903 | 15452 | 258 | 128 | 40 | CC | GG |
| 0904 | 15294 | 259 | 59 | 16 | CC | GG |
| 0905 | 12623 | 268 | 50 | 30 | CC | GG |
| 0906 | 13631 | 274 | 70 | 25 | CC | GG |
| 0907 | 13287 | 299 | 63 | 2 | CC | GG |
| 0908 | 13573 | 300 | 57 | 1 | CT | CG |
| 0909 | 12558 | 276 | 80 | 23 | CC | GG |
| 0926 | 12981 | 254 | 58 | 39 | CC | GG |
| 0927 | 14411 | 278 | 102 | 20 | CT | CG |
| 0928 | 15655 | 286 | 60 | 14 | CC | GG |
| 0929 | 12361 | 307 | 32 | 0 | CT | CG |
| 0930 | 10554 | 254 | 133 | 47 | CC | GG |
| 0932 | 10865 | 282 | 75 | 8 | CC | GG |
| 1086 | 10915 | 274 | 48 | 26 | CC | GG |
| 1097 | 12585 | 256 | 94 | 43 | CC | GG |
| 1103 | 11578 | 267 | 75 | 30 | CC | GG |
| 1104 | 14560 | 291 | 77 | 9 | CC | GG |
| 1106 | 12382 | 252 | 65 | 41 | CT | CG |
| 1108 | 13344 | 283 | 64 | 5 | CT | CG |
| 1109 | 15543 | 293 | 80 | 8 | CC | GG |
| 1110 | 15190 | 290 | 62 | 10 | CC | GG |
| 1111 | 11472 | 288 | 54 | 5 | CC | GG |
| 1112 | 11772 | 264 | 69 | 32 | CC | GG |
| 1113 | 10864 | 297 | 40 | 2 | CT | CG |
| 1114 | 10761 | 262 | 115 | 36 | CC | GG |
| 1116 | 13130 | 253 | 95 | 40 | CT | CG |
| 1117 | 15212 | 273 | 120 | 27 | CC | GG |
| 1118 | 13740 | 300 | 42 | 1 | CT | CG |
| 1120 | 15682 | 285 | 94 | 13 | CT | CG |
| 1121 | 10618 | 275 | 83 | 24 | CC | GG |
| 1192 | 15761 | 297 | 36 | 4 | TT | GG |
| 1193 | 14666 | 266 | 72 | 14 | CC | GG |
| 1194 | 14939 | 280 | 60 | 16 | CC | GG |
| 1195 | 09794 | 253 | 113 | 38 | CT | CG |
| 1196 | 14412 | 286 | 77 | 12 | CT | CG |
| 1197 | 13998 | 301 | 45 | 0 | CC | GG |
| 1199 | 15251 | 269 | 54 | 29 | CC | GG |
| 1200 | 13243 | 293 | 53 | 8 | CT | CG |
| 1201 | 10757 | 271 | 67 | 26 | CC | GG |
| 1202 | 14009 | 288 | 84 | 13 | TT | CC |
| 1203 | 14900 | 267 | 88 | 27 | CC | GG |
| 1205 | 09952 | 288 | 103 | 12 | CC | GG |
| 1206 | 15350 | 267 | 46 | 20 | CC | GG |
| 1208 | 14639 | 269 | 53 | 32 | CC | GG |
| 1209 | 12730 | 278 | 59 | 21 | CT | CG |
| 1210 | 10349 | 269 | 62 | 25 | CC | GG |
| 1211 | 13265 | 263 | 108 | 30 | CT | CG |
| 1212 | 11193 | 291 | 85 | 9 | CT | CG |
| 1213 | 11761 | 253 | 66 | 41 | CC | GG |
| 1214 | 12371 | 276 | 71 | 23 | CT | CG |
| 1215 | 11081 | 273 | 107 | 27 | CT | CG |
| 1216 | 13811 | 299 | 55 | 2 | CC | GG |
| 1217 | 11219 | 290 | 35 | 9 | CC | GG |
| 1218 | 12310 | 273 | 34 | 13 | CC | GG |
| 1220 | 15758 | 254 | 82 | 44 | CT | CG |
| 1221 | 14178 | 270 | 89 | 25 | CT | CG |
| 1222 | 10510 | 276 | 88 | 24 | CC | GG |
| 1223 | 12390 | 349 | 32 | 0 | CT | CG |
| 1224 | 12005 | 300 | 33 | 1 | CC | GG |
| 1225 | 11358 | 256 | 62 | 16 | CT | CG |
| 1226 | 14557 | 282 | 79 | 12 | CC | CG |
| 1227 | 13692 | 285 | 107 | 11 | CC | GG |
| 1228 | 15604 | 255 | 96 | 40 | CC | GG |
| 1229 | 12486 | 251 | 105 | 39 | CC | GG |
| 1230 | 10197 | 254 | 92 | 14 | CT | CG |
| 1324 | 13614 | 303 | 41 | 0 | CT | CG |
| 1325 | 14846 | 259 | 102 | 40 | CT | CG |
| 1326 | 13197 | 280 | 76 | 15 | CT | CG |
| 1327 | 11346 | 277 | 90 | 22 | CT | CG |
| 1328 | 13309 | 271 | 95 | 21 | CT | CG |
| 1329 | 12163 | 281 | 57 | 16 | CC | GG |
| 1330 | 14535 | 286 | 71 | 7 | CC | GG |
| 1331 | 09975 | 261 | 56 | 26 | CC | GG |
| 1333 | 11832 | 277 | 122 | 21 | CC | GG |
| 1334 | 14343 | 298 | 51 | 3 | CC | GG |
| 1337 | 12333 | 296 | 67 | 5 | CC | GG |
| 1338 | 12933 | 282 | 64 | 19 | CC | GG |
| 1339 | 15487 | 273 | 102 | 26 | CT | CG |
| 1341 | 13214 | 298 | 41 | 2 | CC | GG |
| 1342 | 13794 | 277 | 50 | 22 | CT | CG |
| 1343 | 11931 | 264 | 51 | 33 | CC | GG |
| 1344 | 13618 | 292 | 105 | 9 | CC | GG |
| 1345 | 15381 | 253 | 76 | 17 | CC | GG |
| 1346 | 12907 | 240 | 101 | 43 | CT | CG |
| 1348 | 13200 | 269 | 81 | 24 | CC | GG |
| 1349 | 12179 | 263 | 76 | 8 | TT | GG |
| 1350 | 12617 | 253 | 126 | 42 | CT | CG |
| 1351 | 11183 | 301 | 37 | 0 | CT | CG |
| 1352 | 13870 | 287 | 104 | 10 | CC | GG |
| 1353 | 15352 | 288 | 64 | 8 | CC | GG |
| 1355 | 15433 | 252 | 57 | 44 | CT | CG |
| 1357 | 13889 | 267 | 86 | 32 | CT | CG |
| 1358 | 13867 | 294 | 62 | 6 | CC | GG |
| 1359 | 14357 | 292 | 50 | 9 | CC | GG |
| 1361 | 11670 | 269 | 123 | 29 | CT | CG |
| 1363 | 11251 | 263 | 42 | 28 | CT | CG |
| 1364 | 12105 | 280 | 80 | 16 | CC | GG |
| 1454 | 12519 | 263 | 133 | 36 | CC | GG |
| 1455 | 10095 | 265 | 120 | 32 | CT | CG |
| 1456 | 13329 | 285 | 49 | 11 | CC | GG |
| 1457 | 11804 | 271 | 78 | 28 | CC | GG |
| 1458 | 13361 | 255 | 77 | 40 | CC | GG |
| 1460 | 12883 | 255 | 111 | 40 | CT | CG |
| 1463 | 13068 | 279 | 58 | 13 | CC | GG |
| 1464 | 13874 | 279 | 70 | 18 | CC | GG |
| 1465 | 15690 | 277 | 68 | 18 | CC | GG |
| 1466 | 15210 | 249 | 114 | 47 | CC | GG |
| 1467 | 15250 | 297 | 89 | 4 | CC | GG |
| 1468 | 11184 | 276 | 79 | 21 | CT | CG |
| 1469 | 15654 | 279 | 92 | 20 | CC | GG |
| 1470 | 11265 | 279 | 66 | 16 | CT | CG |
| 1472 | 10747 | 247 | 93 | 47 | CC | GG |
| 1474 | 11255 | 294 | 40 | 7 | TT | CC |
| 1476 | 10776 | 316 | 81 | 0 | CC | GG |
| 1477 | 11532 | 288 | 100 | 17 | TT | CC |
| 1478 | 15399 | 257 | 76 | 42 | CT | CG |
| 1479 | 12565 | 277 | 89 | 16 | CC | GG |
| 1481 | 12274 | 277 | 59 | 23 | CT | CG |
| 1483 | 11344 | 285 | 59 | 15 | CC | GG |
| 1484 | 15883 | 277 | 67 | 15 | CC | GG |
| 1485 | 10304 | 312 | 58 | 0 | CT | CG |
| 1486 | 12177 | 269 | 98 | 31 | CT | CG |
| 1488 | 13371 | 266 | 80 | 31 | CC | GG |
| 1489 | 14067 | 250 | 60 | 23 | CC | GG |
| 1490 | 13115 | 250 | 110 | 21 | CT | CG |
| 1492 | 14708 | 250 | 139 | 49 | CC | GG |
| 1493 | 11682 | 285 | 54 | 12 | CC | GG |
| 1494 | 12028 | 270 | 82 | 28 | CT | CG |
| 1495 | 12768 | 253 | 120 | 45 | CT | CG |
| 1609 | 16806 | 308 | 74 | 0 | CT | CG |
| 1611 | 21905 | 282 | 82 | 18 | CC | GG |
| 1612 | 18309 | 299 | 88 | 1 | CC | GG |
| 1616 | 19323 | 255 | 88 | 38 | CT | CG |
| 1618 | 21451 | 292 | 57 | 7 | CT | CG |
| 1621 | 21196 | 270 | 52 | 18 | CC | GG |
| 1622 | 20357 | 270 | 92 | 28 | CT | CG |
| 1623 | 18986 | 268 | 45 | 23 | CC | GG |
| 1626 | 16097 | 284 | 43 | 15 | CC | GG |
| 1628 | 21502 | 278 | 59 | 19 | CT | CG |
| 1629 | 17856 | 290 | 40 | 8 | CT | CG |
| 1630 | 20857 | 271 | 56 | 25 | CC | GG |
| 1632 | 21797 | 272 | 76 | 23 | CC | GG |
| 1635 | 16137 | 285 | 54 | 13 | CC | GG |
| 1639 | 19983 | 299 | 65 | 1 | CC | GG |
| 1640 | 19582 | 336 | 56 | 3 | TT | CC |
| 1642 | 15967 | 288 | 88 | 10 | CT | CG |
| 1643 | 20262 | 297 | 76 | 2 | CT | CG |
| 1648 | 20461 | 280 | 58 | 1 | CC | GG |
| 1649 | 20282 | 280 | 70 | 15 | CC | GG |
| 1653 | 16300 | 270 | 60 | 24 | CC | GG |
| 1655 | 18693 | 280 | 44 | 1 | CT | CG |
| 1656 | 21292 | 280 | 79 | 2 | CC | GG |
| 1663 | 18064 | 272 | 92 | 26 | CT | CG |
| 1665 | 19100 | 289 | 43 | 10 | TT | CC |
| 1734 | 21638 | 284 | 92 | 12 | CC | GG |
| 1736 | 18744 | 245 | 66 | 47 | CC | GG |
| 1738 | 17414 | 272 | 73 | 22 | CT | CG |
| 1740 | 17665 | 271 | 45 | 15 | CC | GG |
| 1745 | 18399 | 279 | 110 | 18 | CC | GG |
| 1749 | 19722 | 284 | 48 | 13 | CT | CG |
| 1750 | 16926 | 289 | 84 | 11 | CC | GG |
| 1751 | 17597 | 278 | 49 | 20 | CC | GG |
| 1758 | 16798 | 282 | 67 | 14 | CC | GG |
| 1762 | 21197 | 252 | 80 | 29 | CC | CG |
| 1764 | 20052 | 303 | 54 | 0 | CC | GG |
| 1767 | 17284 | 282 | 52 | 13 | CC | GG |
| 1771 | 18426 | 300 | 70 | 0 | CT | CG |
| 1775 | 16938 | 258 | 40 | 32 | CT | CG |
| 1779 | 17866 | 282 | 80 | 18 | CC | GG |
| 1780 | 17380 | 287 | 39 | 13 | TT | CC |
| 1781 | 19873 | 280 | 38 | 16 | CC | GG |
| 1782 | 18260 | 285 | 78 | 14 | CC | GG |
| 1786 | 17404 | 296 | 66 | 4 | CC | GG |
| 1787 | 20910 | 270 | 37 | 26 | CC | GG |
| 1789 | 18328 | 280 | 31 | 15 | CC | GG |
| 1790 | 21909 | 288 | 91 | 12 | CT | CG |
| 1791 | 20253 | 320 | 59 | 0 | CT | CG |
| 1792 | 18752 | 292 | 68 | 8 | CC | GG |
| 1797 | 19571 | 286 | 47 | 14 | CC | GG |
| 1798 | 19496 | 293 | 37 | 7 | CT | CG |
| 1801 | 17736 | 252 | 115 | 45 | CT | CG |
| 1802 | 20540 | 258 | 84 | 35 | CC | GG |
| 1804 | 17447 | 269 | 78 | 30 | CC | GG |
| 1809 | 17833 | 252 | 84 | 48 | CC | GG |
| 1812 | 20147 | 270 | 108 | 26 | CT | CG |
| 1813 | 21783 | 270 | 91 | 28 | CT | CG |
| 1818 | 22047 | 253 | 132 | 42 | CC | GG |
| 1820 | 16689 | 268 | 66 | 27 | CC | GG |
| 1822 | 20896 | 271 | 104 | 29 | CC | GG |
| 1825 | 19591 | 263 | 77 | 36 | CT | CG |
| 1828 | 20352 | 271 | 86 | 28 | CT | CG |
| 1832 | 21552 | 248 | 80 | 49 | CC | GG |
| 1835 | 17602 | 278 | 61 | 22 | CT | CG |
| 1838 | 22002 | 273 | 102 | 26 | CT | CG |
| 1839 | 20733 | 268 | 126 | 30 | CC | GG |
| 1840 | 20708 | 248 | 111 | 50 | CT | CG |
| 1844 | 19669 | 259 | 106 | 37 | CT | CG |
| 1845 | 18908 | 267 | 67 | 30 | CT | CG |
| 1847 | 21356 | 271 | 109 | 29 | CT | CG |
| 1849 | 20829 | 262 | 62 | 37 | CC | GG |
| 1852 | 18980 | 267 | 77 | 31 | CT | CG |
| 1854 | 18593 | 272 | 71 | 27 | CC | GG |
| 1855 | 19498 | 261 | 76 | 33 | CC | GG |
| 1857 | 19529 | 254 | 115 | 43 | CC | GG |
| 1859 | 19482 | 267 | 73 | 29 | CT | CG |
| 1861 | 20947 | 249 | 115 | 46 | CC | GG |
| 1862 | 17429 | 243 | 116 | 57 | CT | CG |
| 1863 | 21611 | 258 | 96 | 37 | CT | CG |
| 1873 | 20751 | 246 | 124 | 53 | CT | CG |
| 1874 | 19296 | 257 | 63 | 39 | CC | GG |
| 1876 | 21105 | 267 | 78 | 30 | CC | GG |
| 1877 | 19122 | 264 | 38 | 33 | CT | CG |
| 1878 | 21179 | 267 | 60 | 29 | CT | CG |
| 1879 | 20445 | 278 | 39 | 3 | TT | CC |
| 1880 | 20038 | 259 | 40 | 32 | CC | GG |
| 1881 | 20310 | 288 | 44 | 7 | CC | GG |
| 1882 | 18905 | 270 | 95 | 26 | CC | GG |
| 1883 | 16994 | 279 | 63 | 20 | CC | GG |
| 1884 | 17658 | 255 | 105 | 43 | CC | GG |
| 1885 | 19750 | 280 | 41 | 16 | CT | CG |
| 1886 | 20690 | 277 | 35 | 18 | CT | GG |
| 1890 | 21237 | 252 | 103 | 45 | CT | CG |
| 1896 | 16679 | 283 | 64 | 14 | CT | CG |
| 1899 | 19531 | 272 | 33 | 26 | CC | GG |
| 1900 | 19685 | 272 | 70 | 22 | CT | CG |
| 1901 | 18405 | 272 | 84 | 24 | CT | CG |
| 1904 | 16421 | 298 | 52 | 2 | CC | GG |
| 1905 | 15982 | 267 | 50 | 18 | CT | CG |
| 1906 | 19935 | 272 | 69 | 28 | CC | GG |
| 1914 | 18480 | 290 | 80 | 10 | CT | CG |
| 1915 | 18711 | 260 | 40 | 27 | CC | GG |
| 1919 | 20372 | 255 | 45 | 29 | CT | CG |
| 1921 | 18115 | 294 | 44 | 6 | CC | GG |
| 1922 | 20381 | 298 | 31 | 2 | CC | GG |
| 1924 | 18373 | 277 | 78 | 23 | CC | GG |
| 1925 | 16702 | 295 | 45 | 3 | CT | CG |
| 1926 | 20361 | 280 | 50 | 18 | CC | GG |
| 1929 | 17637 | 278 | 71 | 21 | CC | GG |
| 2007 | 21038 | 286 | 68 | 12 | CC | GG |
| 2011 | 17812 | 279 | 85 | 18 | CT | CG |
| 2012 | 18859 | 275 | 44 | 20 | CC | GG |
| 2013 | 20258 | 288 | 72 | 11 | CT | CG |
| 2015 | 17711 | 278 | 86 | 19 | CC | GG |
| 2016 | 19021 | 285 | 74 | 13 | CC | GG |
| 2018 | 18690 | 263 | 37 | 31 | CT | CG |
| 2020 | 18573 | 280 | 69 | 16 | CC | GG |
| 2021 | 19445 | 284 | 36 | 13 | CT | CG |
| 2024 | 20313 | 288 | 45 | 9 | CC | GG |
| 2025 | 21506 | 280 | 31 | 15 | CC | GG |
| 2027 | 18692 | 291 | 32 | 9 | CC | GG |
| 2028 | 21713 | 312 | 31 | 1 | TT | CC |
| 2029 | 17016 | 274 | 39 | 24 | CC | GG |
| 2031 | 19742 | 270 | 72 | 30 | CT | CG |
| 2033 | 20922 | 276 | 56 | 17 | CT | CG |
| 2036 | 20279 | 283 | 93 | 15 | CC | GG |
| 2040 | 19553 | 303 | 76 | 2 | TT | GG |
| 2044 | 16299 | 289 | 36 | 9 | CC | GG |
| 2045 | 16890 | 269 | 78 | 25 | CT | CG |
| 2048 | 19414 | 269 | 38 | 23 | CC | GG |
| 2049 | 20421 | 293 | 50 | 7 | CT | CG |
| 2053 | 17709 | 256 | 45 | 1 | CC | GG |
| 2054 | 19049 | 286 | 34 | 14 | TT | CC |
| 2058 | 19863 | 263 | 92 | 37 | CC | GG |
| 2061 | 20003 | 316 | 50 | 0 | CT | CG |
| 2063 | 19071 | 297 | 65 | 3 | CC | GG |
| 2064 | 17166 | 285 | 50 | 12 | CT | CG |
| 2067 | 17565 | 280 | 81 | 20 | CC | GG |
| 2141 | 19611 | 241 | 40 | 40 | CC | GG |
| 2143 | 20102 | 275 | 97 | 17 | CC | GG |
| 2145 | 16094 | 275 | 53 | 22 | CT | CG |
| 2146 | 20864 | 275 | 80 | 19 | CT | CG |
| 2152 | 19334 | 281 | 69 | 19 | CC | GG |
| 2158 | 19501 | 277 | 42 | 22 | CC | GG |
| 2159 | 16493 | 301 | 41 | 0 | CT | CG |
| 2160 | 18953 | 285 | 44 | 15 | CT | CG |
| 2164 | 20604 | 271 | 47 | 29 | CC | GG |
| 2169 | 17620 | 268 | 33 | 32 | CT | GG |
| 2170 | 19472 | 290 | 57 | 10 | CC | GG |
| 2172 | 19890 | 305 | 57 | 0 | CC | GG |
| 2174 | 19794 | 298 | 59 | 2 | CT | CG |
| 2176 | 17807 | 336 | 35 | 0 | CT | CG |
| 2178 | 16117 | 317 | 44 | 0 | CT | CG |
| 2183 | 21236 | 283 | 56 | 15 | CT | CG |
| 2186 | 21262 | 259 | 56 | 30 | CC | GG |
| 2189 | 17676 | 300 | 60 | 0 | CC | GG |
| 2190 | 19719 | 284 | 43 | 14 | CT | CG |
| 2193 | 17102 | 301 | 61 | 0 | CT | CG |
| 2195 | 17056 | 274 | 37 | 3 | CC | GG |
| 2196 | 17877 | 274 | 68 | 2 | CC | GG |
| 2200 | 22023 | 294 | 40 | 5 | CT | CG |
| 2308 | 17592 | 237 | 153 | 62 | CT | CG |
| 2309 | 21839 | 270 | 68 | 27 | CC | GG |
| 2311 | 17735 | 263 | 102 | 34 | CT | CG |
| 2312 | 21582 | 244 | 96 | 53 | CC | GG |
| 2314 | 21224 | 270 | 111 | 27 | CT | CG |
| 2321 | 19365 | 247 | 105 | 51 | CC | GG |
| 2323 | 21138 | 258 | 53 | 35 | CC | GG |
| 2328 | 19811 | 264 | 111 | 30 | CC | GG |
| 2330 | 20854 | 262 | 135 | 35 | CC | GG |
| 2332 | 20239 | 274 | 81 | 21 | CT | CG |
| 2333 | 21563 | 258 | 58 | 40 | CC | GG |
| 2334 | 17010 | 278 | 33 | 14 | CC | GG |
| 2335 | 20901 | 276 | 51 | 15 | CC | GG |
| 2336 | 19437 | 293 | 69 | 6 | CT | CG |
| 2338 | 16783 | 297 | 38 | 2 | TT | GG |
| 2339 | 20014 | 270 | 111 | 28 | CT | CG |
| 2341 | 19080 | 258 | 82 | 32 | CC | GG |
| 2342 | 18320 | 284 | 44 | 13 | CC | GG |
| 2344 | 19275 | 271 | 39 | 19 | CT | GG |
| 2345 | 17677 | 279 | 78 | 21 | CC | GG |
| 2346 | 16805 | 280 | 108 | 16 | CC | GG |
| 2347 | 19855 | 265 | 65 | 30 | CC | GG |
| 2349 | 21617 | 291 | 67 | 9 | CC | GG |
| 2352 | 19972 | 278 | 38 | 17 | TT | CC |
| 2353 | 17087 | 335 | 51 | 0 | CT | CG |
| 2355 | 19084 | 268 | 67 | 20 | CC | GG |
| 2357 | 20032 | 292 | 77 | 8 | CT | CG |
| 2358 | 21653 | 239 | 80 | 47 | CC | GG |
| 2359 | 21001 | 270 | 104 | 25 | CC | GG |
| 2360 | 20828 | 282 | 103 | 19 | CC | GG |
| 2361 | 16125 | 269 | 60 | 29 | CC | GG |
| 2363 | 21171 | 249 | 132 | 51 | CC | GG |
| 2364 | 18087 | 283 | 72 | 10 | CC | GG |
| 2365 | 20093 | 258 | 43 | 24 | CT | CG |
| 2366 | 17855 | 286 | 52 | 11 | CC | GG |
| 2368 | 19541 | 317 | 69 | 4 | TT | CC |
| 2368 | 19541 | 317 | 69 | 5 | TT | CC |
| 2369 | 21483 | 265 | 34 | 23 | CC | GG |
| 2369 | 21483 | 265 | 34 | 23 | CC | GG |
| 2370 | 19783 | 256 | 135 | 42 | CC | CG |
| 2371 | 20716 | 243 | 120 | 55 | CC | GG |
| 2372 | 16487 | 245 | 148 | 49 | CC | GG |
| 2373 | 21850 | 261 | 121 | 31 | CC | GG |
| 2375 | 17285 | 257 | 89 | 32 | CC | GG |
| 2376 | 21364 | 278 | 51 | 18 | CC | GG |
| 2376 | 21364 | 278 | 51 | 18 | CC | GG |
| 2377 | 20946 | 248 | 120 | 48 | CC | GG |
| 2378 | 16775 | 272 | 88 | 19 | CC | GG |
| 2378 | 16775 | 272 | 88 | 19 | CC | GG |
| 2379 | 20717 | 259 | 91 | 35 | CC | GG |
| 2380 | 22042 | 262 | 99 | 32 | CC | GG |
| 2382 | 18360 | 278 | 52 | 21 | CC | GG |
| 2382 | 18360 | 278 | 52 | 21 | CC | GG |
| 2383 | 21701 | 258 | 90 | 38 | CC | GG |
| 2385 | 20440 | 257 | 132 | 42 | CC | CG |
| 2386 | 21556 | 257 | 65 | 34 | CT | CG |
| 2387 | 17194 | 267 | 74 | 23 | CC | GG |
| 2387 | 17194 | 267 | 74 | 23 | CC | GG |
| 2389 | 19709 | 290 | 64 | 9 | CT | CG |
| 2389 | 19709 | 290 | 64 | 9 | CT | CG |
| 2390 | 17217 | 274 | 68 | 10 | CC | GG |
| 2390 | 17217 | 274 | 68 | 10 | CC | GG |
| 2391 | 19356 | 254 | 137 | 41 | CC | GG |
| 2392 | 20815 | 276 | 62 | 17 | CC | GG |
| 2392 | 20815 | 276 | 62 | 17 | CC | GG |
| 2394 | 21733 | 249 | 144 | 55 | CT | CG |
| 2395 | 18406 | 253 | 138 | 43 | CC | GG |
| 2401 | 18734 | 265 | 90 | 29 | CT | CG |
| 2402 | 17864 | 286 | 62 | 13 | CC | GG |
| 2404 | 16991 | 285 | 98 | 11 | CC | GG |
| 2406 | 17698 | 269 | 120 | 27 | CC | GG |
| 2407 | 16131 | 294 | 42 | 5 | CC | GG |
| 2408 | 18397 | 262 | 66 | 32 | CC | GG |
| 2409 | 16816 | 267 | 33 | 33 | CC | GG |
| 2410 | 18539 | 267 | 88 | 32 | CC | GG |
| 2411 | 16511 | 320 | 46 | 0 | CT | CG |
| 2412 | 20957 | 285 | 94 | 15 | TT | CC |
| 2413 | 18900 | 288 | 50 | 9 | CC | GG |
| 2414 | 19874 | 288 | 36 | 10 | CC | GG |
| 2419 | 20467 | 290 | 76 | 10 | CT | CG |
| 2426 | 21360 | 261 | 37 | 31 | CC | GG |
| 2427 | 18956 | 304 | 45 | 0 | CT | CG |
| 2431 | 16973 | 285 | 63 | 14 | CC | GG |
| 2433 | 21418 | 287 | 45 | 11 | CC | GG |
| 2436 | 22057 | 271 | 58 | 18 | CC | GG |
| 2439 | 18046 | 284 | 90 | 15 | CC | GG |
| 2440 | 19826 | 287 | 86 | 13 | CT | CG |
| 2443 | 19751 | 283 | 33 | 15 | CT | CG |
| 2445 | 20247 | 288 | 107 | 15 | TT | CC |
| 2446 | 17057 | 284 | 74 | 13 | CC | GG |
| 2451 | 18983 | 290 | 66 | 12 | TT | CC |
| 2452 | 20995 | 255 | 54 | 45 | CT | CG |
| 2454 | 19108 | 284 | 65 | 15 | CT | CG |
| 2458 | 18334 | 257 | 31 | 31 | CT | CG |
| 2459 | 16533 | 335 | 61 | 7 | TT | CC |
| 2463 | 17883 | 286 | 82 | 14 | CC | GG |
| 2464 | 21186 | 290 | 52 | 9 | CT | CG |
| 2465 | 17743 | 266 | 36 | 19 | CC | GG |
| 2468 | 17165 | 291 | 65 | 9 | CT | CG |
| 2470 | 16504 | 296 | 42 | 4 | CC | GG |
| 2529 | 19483 | 294 | 39 | 5 | TT | GG |
| 2533 | 20625 | 309 | 77 | 7 | TT | CC |
| 2538 | 18653 | 262 | 66 | 30 | CC | GG |
| 2542 | 16641 | 278 | 76 | 19 | CT | CG |
| 2543 | 19029 | 302 | 78 | 1 | TT | GG |
| 2547 | 20951 | 251 | 88 | 38 | CC | GG |
| 2552 | 19530 | 251 | 41 | 34 | CC | GG |
| 2556 | 22113 | 270 | 80 | 25 | CT | CG |
| 2557 | 18127 | 285 | 59 | 15 | CC | GG |
| 2560 | 20105 | 295 | 47 | 4 | CC | GG |
| 2562 | 21471 | 291 | 79 | 9 | CT | CG |
| 2563 | 18103 | 285 | 46 | 14 | CC | GG |
| 2566 | 16655 | 288 | 36 | 11 | CT | CG |
| 2567 | 19315 | 268 | 71 | 23 | CC | GG |
| 2569 | 21379 | 282 | 38 | 17 | CC | GG |
| 2572 | 20280 | 284 | 50 | 13 | CC | GG |
| 2574 | 18374 | 270 | 41 | 30 | CT | CG |
| 2575 | 19903 | 291 | 46 | 9 | CC | GG |
| 2576 | 20945 | 286 | 48 | 13 | CC | CG |
| 2577 | 18756 | 291 | 50 | 8 | CC | GG |
| 2586 | 21676 | 251 | 62 | 38 | CC | GG |
| 2895 | 21097 | 249 | 65 | 38 | CT | CG |
| 2896 | 17728 | 251 | 100 | 39 | CC | GG |
| 2902 | 21482 | 267 | 86 | 31 | CT | CG |
| 2906 | 20402 | 273 | 98 | 22 | CT | CG |
| 2907 | 21414 | 247 | 97 | 45 | CC | GG |
| 2912 | 20221 | 256 | 72 | 36 | CC | GG |
| 2918 | 21890 | 239 | 95 | 45 | CC | GG |
| 2919 | 17389 | 252 | 131 | 46 | CC | CG |
| 2920 | 20083 | 267 | 78 | 32 | CC | GG |
| 2921 | 15968 | 250 | 123 | 44 | CC | GG |
| 2923 | 20135 | 254 | 116 | 45 | CC | GG |
| 2929 | 20743 | 258 | 96 | 37 | CC | GG |
| 2934 | 19927 | 261 | 67 | 34 | CT | CG |
| 2937 | 17696 | 264 | 108 | 33 | CC | GG |
| 2939 | 17020 | 262 | 80 | 30 | CC | GG |
| 2948 | 21263 | 255 | 109 | 41 | CC | GG |
| 2950 | 18748 | 264 | 113 | 36 | CC | GG |
| 2958 | 19422 | 261 | 119 | 35 | CT | CG |
| 2959 | 20263 | 270 | 121 | 29 | CT | CG |
| 2961 | 17334 | 251 | 98 | 40 | CC | GG |
| 2964 | 17802 | 258 | 97 | 27 | CT | CG |
| 2964 | 17802 | 258 | 97 | 27 | CT | CG |
| 2969 | 16400 | 257 | 110 | 41 | CT | CG |
| 2974 | 20822 | 266 | 101 | 27 | CC | GG |
| 2985 | 20148 | 255 | 82 | 31 | CT | CG |
| 2988 | 17435 | 255 | 106 | 38 | CT | CG |
| 2991 | 17857 | 249 | 83 | 41 | CT | CG |
| 3050 | 18626 | 255 | 108 | 44 | CT | CG |
| 3054 | 20693 | 247 | 86 | 49 | CC | GG |
| 3055 | 20750 | 243 | 138 | 57 | CT | CG |
| 3057 | 20137 | 244 | 97 | 54 | CC | GG |
| 3058 | 19697 | 258 | 86 | 40 | CC | GG |
| 3059 | 16709 | 273 | 102 | 25 | TT | CC |
| 3062 | 21606 | 245 | 75 | 28 | CC | GG |
| 3063 | 19587 | 245 | 80 | 35 | CC | GG |
| 3065 | 20712 | 242 | 136 | 53 | CC | GG |
| 3066 | 18549 | 268 | 96 | 32 | CC | GG |
| 3067 | 22048 | 237 | 125 | 58 | CC | GG |
| 3068 | 20574 | 266 | 61 | 25 | CT | CG |
| 3073 | 19304 | 255 | 75 | 40 | CT | CG |
| 3074 | 17438 | 266 | 67 | 30 | CT | CG |
| 3075 | 16891 | 248 | 96 | 48 | CC | GG |
| 3083 | 15966 | 254 | 129 | 44 | CT | CG |
| 3085 | 17700 | 269 | 96 | 31 | CC | GG |
| 3089 | 20454 | 268 | 84 | 31 | CC | GG |
| 3092 | 21226 | 260 | 61 | 40 | CC | GG |
| 3093 | 17801 | 270 | 99 | 24 | CT | CG |
| 3094 | 19485 | 246 | 112 | 55 | CC | GG |
| 3099 | 21230 | 249 | 107 | 46 | CT | CG |
| 3110 | 20934 | 252 | 105 | 41 | CT | CG |
| 3111 | 20900 | 263 | 68 | 31 | CC | GG |
| 3113 | 16849 | 260 | 99 | 34 | CC | GG |
| 3114 | 20879 | 249 | 105 | 51 | CT | CG |
| 3117 | 20325 | 267 | 92 | 29 | CC | GG |
| 3118 | 21554 | 248 | 137 | 51 | CT | CG |

**Notes.**

CAGE=cage number; cx_wb=wing number; FEA = first egg age; E59W = egg number at age 59 weeks; E300D = egg number at age 300 days.

**Supplementary table S4 Raw data of association at DRD2 gene**

| CAGE | cx_wb | FEA | E59W | E300D | T+3024C | A+3183C | A+3262G | C+3301G | T+3423C | T+3428C | A+3484T | A+3489G | C+3545G |
| --- | --- | --- | --- | --- | --- | --- | --- | --- | --- | --- | --- | --- | --- |
| 0022 | 11280 | 290 | 38 | 8 | TC | AA | AA | CC | CC | CC | AA | AA | CC |
| 0023 | 13211 | 294 | 72 | 7 | TC | CC | GG | CC | TC | CC | AA | AG | CC |
| 0024 | 13734 | 275 | 60 | 22 | TT | AC | AG | CG | TC | CC | AA | AG | CG |
| 0025 | 13954 | 262 | 69 | 25 | CC | CC | GG | CC | TT | CC | AA | GG | CC |
| 0026 | 12173 | 276 | 55 | 20 | CC | AC | GG | CG | TC | CC | AT | AG | CG |
| 0027 | 13487 | 281 | 45 | 18 | CC | CC | GG | CC | TT | CC | AA | GG | CC |
| 0028 | 13491 | 290 | 63 | 11 | CC | AA | GG | CC | TC | CC | AA | GG | CC |
| 0029 | 13430 | 343 | 50 | 0 | CC | AA | AA | CC | CC | TC | AA | AA | CC |
| 0030 | 13599 | 276 | 57 | 20 | CC | AA | AG | CG | TC | TC | AA | AG | CC |
| 0031 | 12953 | 240 | 150 | 49 | CC | AC | GG | CG | TC | CC | AT | AG | CG |
| 0032 | 11954 | 307 | 37 | 0 | TC | AA | AA | CC | CC | CC | AA | AA | CC |
| 0033 | 14285 | 296 | 45 | 5 | CC | CC | GG | CC | TT | CC | AA | GG | CC |
| 0035 | 14561 | 298 | 90 | 2 | CC | AA | AG | CG | TC | TC | AA | AA | CC |
| 0036 | 10100 | 298 | 68 | 3 | CC | AA | AA | CC | CC | TC | AA | GG | CC |
| 0037 | 15511 | 275 | 80 | 24 | CC | AA | AG | CC | CC | TC | AA | AA | CC |
| 0038 | 14207 | 292 | 68 | 8 | TC | AC | AG | CG | TC | CC | AT | AG | CC |
| 0039 | 15512 | 259 | 74 | 37 | TC | AA | AA | CC | CC | TC | AA | AA | CC |
| 0040 | 15385 | 315 | 47 | 0 | CC | AC | GG | CG | TC | CC | AA | AG | CG |
| 0045 | 15469 | 265 | 103 | 29 | TC | AC | AG | CG | TC | CC | AT | AG | CC |
| 0046 | 15233 | 310 | 58 | 0 | TC | AC | AG | CG | TC | CC | AT | AG | CG |
| 0047 | 10723 | 313 | 60 | 0 | TC | AC | AG | CG | TC | CC | AT | AG | CG |
| 0048 | 12288 | 255 | 78 | 33 | TC | AC | AG | CG | TC | CC | AT | AG | CG |
| 0049 | 11054 | 289 | 73 | 12 | CC | AA | AG | CC | TC | CC | AA | AA | CC |
| 0050 | 11685 | 270 | 106 | 26 | TC | AC | AG | CG | TC | CC | AA | AG | CG |
| 0051 | 15306 | 284 | 72 | 17 | TT | AC | AG | CG | TC | CC | AT | AG | CG |
| 0052 | 14405 | 295 | 60 | 4 | TC | AC | AG | CG | TC | CC | AT | AG | CC |
| 0054 | 09840 | 274 | 107 | 27 | CC | AA | AG | CC | CC | CC | AA | AA | CC |
| 0056 | 13063 | 293 | 66 | 8 | TT | AA | AG | CG | TC | CC | AA | AA | CG |
| 0057 | 15392 | 289 | 44 | 12 | CC | CC | GG | CC | TT | CC | AA | GG | CC |
| 0059 | 15068 | 287 | 46 | 13 | CC | AA | AA | CC | CC | TT | AA | AA | CC |
| 0060 | 14633 | 293 | 65 | 8 | TC | AA | AA | CC | CC | TC | AA | AA | CC |
| 0141 | 13743 | 288 | 47 | 9 | TT | AA | AA | CC | CC | CC | AA | AA | CC |
| 0142 | 13674 | 289 | 107 | 12 | CC | AA | GG | CC | CC | CC | AA | AA | CC |
| 0145 | 10254 | 293 | 33 | 6 | TT | AA | AG | CG | TC | CC | AA | AA | CC |
| 0146 | 14985 | 262 | 139 | 37 | CC | AC | AG | CG | TC | CC | AA | AG | CG |
| 0147 | 12395 | 282 | 43 | 18 | TT | AC | AG | CG | TC | CC | AA | AG | CG |
| 0148 | 12085 | 267 | 83 | 31 | CC | AA | GG | CG | TC | CC | AA | AA | CC |
| 0149 | 13687 | 279 | 70 | 22 | CC | AA | AG | CG | TC | TC | AA | AG | CG |
| 0150 | 15596 | 289 | 48 | 5 | CC | CC | GG | CC | TT | CC | AA | GG | CC |
| 0151 | 14867 | 256 | 95 | 43 | TC | AC | AG | CG | TC | CC | AT | AG | CG |
| 0152 | 13736 | 305 | 66 | 0 | TC | AA | AA | CC | CC | CC | AA | GG | CC |
| 0153 | 10353 | 289 | 70 | 12 | TT | AA | AA | CC | CC | CC | AA | AA | CC |
| 0154 | 12379 | 253 | 80 | 42 | CC | CC | GG | CC | TT | CC | AA | GG | CG |
| 0157 | 14071 | 295 | 44 | 5 | CC | CC | GG | CC | TT | CC | AA | GG | CG |
| 0158 | 11689 | 289 | 47 | 10 | CC | CC | GG | CC | TT | CC | AA | GG | CG |
| 0159 | 12172 | 275 | 40 | 14 | TC | AC | AG | CG | TC | CC | AA | AG | CG |
| 0160 | 13454 | 272 | 96 | 26 | CC | AA | AA | CC | TC | CC | AA | AG | CC |
| 0161 | 13907 | 267 | 45 | 29 | TC | AA | AA | CC | CC | TC | AA | AA | CC |
| 0164 | 11466 | 279 | 66 | 22 | CC | AC | AG | CG | TC | TC | AA | AG | CG |
| 0165 | 09934 | 287 | 48 | 14 | TT | AA | AG | CG | TC | CC | AA | AA | CG |
| 0167 | 11822 | 277 | 35 | 20 | CC | AA | GG | CG | TC | CC | AA | AG | CG |
| 0168 | 12574 | 279 | 51 | 13 | TC | AA | AA | CC | CC | TC | AA | AA | CC |
| 0174 | 13919 | 275 | 76 | 24 | CC | AC | GG | CG | TC | CC | AT | AG | CG |
| 0175 | 14365 | 275 | 61 | 21 | CC | AA | AA | CC | CC | CC | AA | AA | CC |
| 0176 | 11478 | 269 | 48 | 27 | TC | AC | AG | CG | TC | CC | AT | AG | CG |
| 0178 | 14659 | 300 | 36 | 1 | TT | AA | AA | CC | CC | CC | AA | AA | CC |
| 0179 | 13297 | 285 | 74 | 11 | TC | AC | AG | CG | TC | CC | AT | AG | CG |
| 0180 | 14231 | 300 | 40 | 1 | CC | AA | AA | CC | CC | TT | AA | AA | CC |
| 0271 | 14488 | 264 | 110 | 27 | TC | AA | AA | CC | CC | TC | AA | AA | CC |
| 0272 | 13720 | 281 | 47 | 1 | TC | AC | AG | CG | TC | CC | AT | AG | CG |
| 0273 | 12255 | 343 | 65 | 0 | CC | AC | GG | CG | TC | CC | AT | AG | CG |
| 0274 | 11985 | 284 | 43 | 12 | CC | CC | GG | CC | TT | CC | AA | GG | CC |
| 0276 | 09769 | 264 | 47 | 24 | CC | AA | AG | CC | CC | CC | AA | AA | CC |
| 0277 | 15010 | 267 | 85 | 31 | CC | AA | AG | CC | CC | TC | AA | AA | CC |
| 0278 | 15708 | 302 | 40 | 0 | TC | AC | AG | CG | TC | CC | AT | AG | CC |
| 0279 | 11313 | 294 | 64 | 7 | TC | AA | AA | CC | CC | CC | AA | AA | CC |
| 0280 | 15829 | 343 | 33 | 0 | CC | AA | AG | CC | CC | TC | AA | AA | CC |
| 0281 | 15588 | 268 | 116 | 31 | CC | AC | AG | CG | TC | TC | AT | AG | CC |
| 0282 | 12687 | 271 | 104 | 25 | CC | AC | AG | CG | TC | TC | AT | AG | CG |
| 0283 | 10926 | 256 | 50 | 28 | CC | CC | GG | CC | TT | CC | AA | GG | CC |
| 0284 | 15109 | 266 | 51 | 34 | TC | AA | AG | CG | TC | CC | AA | AA | CG |
| 0285 | 13096 | 255 | 128 | 41 | CC | AA | AG | CC | TC | TC | AA | AA | GG |
| 0286 | 10265 | 296 | 83 | 5 | CC | AC | GG | CG | TC | CC | AT | AG | CG |
| 0288 | 14756 | 308 | 39 | 0 | CC | AA | AG | CC | CC | CC | AA | AA | CC |
| 0289 | 15351 | 261 | 87 | 30 | CC | AC | AG | CG | TC | TC | AT | AG | CC |
| 0290 | 14988 | 280 | 108 | 21 | TC | AC | AG | CG | TC | TT | AA | AA | CC |
| 0291 | 12531 | 299 | 80 | 2 | CC | CC | GG | CC | TT | CC | AA | GG | CG |
| 0292 | 12321 | 328 | 54 | 0 | TC | AC | AG | CG | TC | CC | AA | AG | CG |
| 0293 | 14570 | 280 | 76 | 13 | TT | AA | AG | CG | TC | CC | AA | AA | CC |
| 0294 | 11023 | 274 | 86 | 19 | CC | CC | GG | CC | TT | CC | AT | AG | CC |
| 0295 | 14205 | 251 | 146 | 46 | CC | AC | AG | CG | TC | TT | AA | AG | CC |
| 0296 | 14596 | 258 | 101 | 39 | TT | AA | AA | CC | CC | CC | AA | AA | CC |
| 0297 | 13321 | 288 | 68 | 12 | CC | CC | GG | CC | TT | CC | AA | GG | CG |
| 0298 | 10783 | 312 | 40 | 0 | CC | AA | AG | CC | CC | TC | AA | AA | CC |
| 0299 | 12978 | 286 | 53 | 11 | TC | AA | AG | CG | TC | CC | AA | AA | CC |
| 0301 | 15843 | 273 | 85 | 23 | TC | AC | AG | CC | TC | CC | AA | AG | CG |
| 0302 | 13204 | 274 | 90 | 17 | CC | AC | AG | CC | TC | TC | AA | AA | CC |
| 0303 | 14111 | 254 | 76 | 42 | CC | CC | GG | CC | TT | CC | AA | GG | CG |
| 0304 | 14301 | 259 | 84 | 30 | CC | AA | AA | CC | CC | TT | AA | AA | CC |
| 0307 | 12199 | 246 | 105 | 46 | CC | CC | GG | CC | TT | CC | AA | AA | CG |
| 0308 | 15033 | 272 | 117 | 25 | TT | AA | AA | CC | CC | TC | AA | AA | GG |
| 0309 | 10889 | 269 | 52 | 29 | CC | CC | GG | CC | TT | CC | AA | GG | CC |
| 0310 | 12351 | 276 | 39 | 21 | CC | CC | GG | CC | TT | CC | AA | GG | CC |
| 0401 | 15536 | 306 | 92 | 0 | TC | AC | AG | CG | TC | CC | AT | AG | CG |
| 0402 | 13882 | 267 | 53 | 29 | TC | AA | AA | CC | CC | TC | AA | AA | CC |
| 0403 | 12697 | 343 | 46 | 0 | TT | AC | AG | CG | TC | CC | AT | AG | CG |
| 0404 | 12063 | 270 | 102 | 28 | CC | CC | GG | CC | TT | CC | AA | GG | CG |
| 0405 | 10338 | 256 | 70 | 40 | TC | AA | AG | CC | CC | CC | AA | AA | CC |
| 0406 | 10201 | 288 | 84 | 10 | TC | AA | AG | CC | CC | CC | AA | AA | CC |
| 0407 | 11246 | 259 | 58 | 27 | CC | CC | GG | CC | TT | CC | AA | GG | CC |
| 0408 | 10337 | 318 | 45 | 0 | TC | AC | AG | CG | TC | CC | AT | AG | CC |
| 0410 | 14358 | 278 | 70 | 16 | TC | AA | AG | CC | CC | CC | AA | AA | CC |
| 0411 | 15314 | 240 | 89 | 42 | TT | AA | AA | CC | CC | CC | AA | AA | CC |
| 0412 | 12602 | 271 | 51 | 26 | TT | AC | AG | CG | TC | CC | AA | AA | CC |
| 0413 | 10417 | 276 | 71 | 21 | CC | CC | GG | CC | TT | CC | AA | GG | CC |
| 0414 | 10873 | 300 | 67 | 1 | TT | AA | AG | CG | TC | CC | AA | AA | CC |
| 0415 | 10282 | 277 | 102 | 23 | TC | AA | AG | CC | CC | CC | AA | AA | CC |
| 0416 | 12997 | 278 | 78 | 21 | CC | AA | AG | CC | CC | CC | AA | AA | CC |
| 0417 | 13150 | 276 | 52 | 22 | TT | AC | AG | CG | TC | CC | AT | AG | CG |
| 0419 | 14496 | 266 | 79 | 35 | CC | CC | GG | CC | TT | CC | AA | GG | CC |
| 0420 | 10803 | 286 | 115 | 15 | CC | AA | AA | CC | CC | TT | AA | AA | CC |
| 0421 | 09959 | 276 | 116 | 22 | TT | AA | AA | CC | CC | TT | AA | AA | GG |
| 0422 | 15540 | 260 | 45 | 37 | CC | AA | AA | CC | CC | TT | AA | AA | CC |
| 0423 | 11136 | 305 | 70 | 0 | CC | AA | AG | CC | TC | CC | AA | AA | CC |
| 0424 | 15448 | 267 | 58 | 21 | TC | AA | AA | CC | CC | CC | AA | AA | CC |
| 0425 | 15909 | 280 | 91 | 17 | CC | CC | GG | CC | TT | CC | AA | GG | CC |
| 0427 | 10268 | 269 | 74 | 32 | TT | AA | AA | CC | CC | CC | AA | AA | CC |
| 0429 | 10371 | 298 | 57 | 3 | TT | AA | AA | CC | CC | CC | AA | AA | CC |
| 0431 | 12514 | 294 | 48 | 5 | CC | CC | GG | CC | TT | CC | AA | GG | CG |
| 0432 | 13022 | 307 | 86 | 0 | CC | AA | AA | CC | CC | CC | AA | AG | CC |
| 0435 | 14817 | 284 | 89 | 17 | TT | AA | AG | CG | TC | TC | AA | AA | GG |
| 0437 | 14727 | 266 | 129 | 32 | CC | AA | AA | CC | CC | TT | AA | AA | CC |
| 0438 | 12817 | 303 | 53 | 0 | CC | CC | GG | CC | TT | CC | AA | GG | CC |
| 0439 | 13336 | 308 | 42 | 0 | TT | AA | AA | CC | CC | CC | AA | AA | CC |
| 0440 | 10961 | 267 | 92 | 28 | CC | AC | AG | CG | TC | TC | AT | AG | CG |
| 0531 | 13152 | 299 | 102 | 2 | CC | AA | GG | CG | TC | CC | AA | AG | CG |
| 0533 | 12383 | 240 | 98 | 50 | TC | AA | AA | CC | TC | CC | AA | AG | CC |
| 0535 | 15030 | 241 | 86 | 30 | TC | AA | AA | CC | CC | CC | AA | AA | CC |
| 0536 | 10046 | 288 | 33 | 13 | CC | AA | GG | CC | CC | CC | AA | AA | CC |
| 0538 | 13276 | 272 | 64 | 28 | CC | AA | AA | CC | CC | TT | AA | AA | CC |
| 0539 | 11718 | 281 | 48 | 16 | TC | AC | AG | CG | TC | CC | AT | AG | CG |
| 0540 | 14294 | 263 | 104 | 35 | CC | AC | AG | CG | TC | CC | AT | AG | CG |
| 0541 | 14396 | 308 | 42 | 0 | CC | AA | GG | CC | CC | CC | AA | AA | CC |
| 0542 | 12811 | 287 | 69 | 14 | TC | AC | AG | CG | TC | CC | AA | AG | CG |
| 0543 | 12525 | 307 | 31 | 0 | TC | AA | AA | CC | CC | TC | AA | AA | CC |
| 0544 | 14699 | 257 | 105 | 34 | CC | AA | GG | CC | CC | CC | AA | AG | CG |
| 0545 | 15635 | 268 | 83 | 28 | CC | AA | AG | CG | TC | CC | AA | AG | CG |
| 0546 | 14083 | 290 | 59 | 10 | CC | AA | AA | CC | CC | TC | AA | AA | CC |
| 0548 | 11893 | 277 | 91 | 17 | TT | AA | AG | CG | TC | CC | AT | AG | CG |
| 0550 | 13223 | 297 | 60 | 4 | TC | AC | AG | CG | TC | CC | AT | AG | CC |
| 0552 | 15558 | 291 | 94 | 10 | TT | AA | AA | CC | CC | CC | AA | AA | CC |
| 0553 | 13131 | 278 | 66 | 23 | CC | CC | GG | CC | TT | CC | AA | GG | CG |
| 0554 | 10327 | 306 | 44 | 0 | TT | AC | AG | CG | TC | CC | AA | AG | CC |
| 0555 | 15329 | 267 | 65 | 24 | TC | AA | AG | CG | TC | CC | AA | AG | CG |
| 0556 | 15567 | 287 | 59 | 12 | CC | AC | GG | CC | CC | CC | AA | AA | CC |
| 0559 | 14855 | 254 | 81 | 43 | TC | AC | AG | CG | TC | CC | AT | AG | CG |
| 0561 | 11628 | 272 | 61 | 20 | CC | AA | AG | CC | CC | TC | AA | AA | CC |
| 0562 | 14075 | 281 | 45 | 15 | CC | AC | GG | CG | TC | CC | AA | AG | CG |
| 0563 | 13972 | 270 | 116 | 30 | CC | AA | AG | CC | CC | TC | AA | AA | CC |
| 0564 | 13989 | 276 | 59 | 23 | CC | AC | AG | CG | TC | TC | AT | AG | CG |
| 0565 | 12599 | 289 | 41 | 12 | CC | AA | AA | CC | CC | TT | AA | AA | CC |
| 0566 | 11281 | 299 | 41 | 2 | TC | AC | AG | CG | TC | CC | AT | AG | CC |
| 0567 | 14656 | 302 | 32 | 0 | CC | AA | GG | CG | TC | CC | AA | AA | CC |
| 0568 | 10198 | 298 | 100 | 3 | TT | AA | AA | CC | CC | CC | AA | AA | CC |
| 0570 | 12951 | 294 | 33 | 7 | TC | AC | AG | CG | TC | CC | AT | AG | CG |
| 0671 | 14436 | 240 | 50 | 40 | TC | AA | AA | CC | CC | CC | AA | AA | CC |
| 0672 | 11865 | 343 | 57 | 0 | TT | AA | AG | CG | TC | CC | AA | AA | CC |
| 0673 | 15601 | 289 | 64 | 10 | TC | AA | AG | CG | TC | CC | AT | AA | CC |
| 0676 | 15528 | 257 | 122 | 41 | TC | AA | AA | CC | CC | TC | AA | AA | GG |
| 0677 | 11339 | 267 | 127 | 30 | CC | AA | AA | CC | CC | TT | AA | AA | CC |
| 0678 | 11017 | 298 | 57 | 2 | CC | AC | GG | CG | TC | CC | AA | AG | CG |
| 0679 | 15623 | 304 | 36 | 0 | CC | AC | GG | CG | TC | CC | AA | AG | CG |
| 0680 | 13603 | 303 | 72 | 0 | CC | AC | AG | CG | TC | TC | AT | AG | CG |
| 0681 | 10284 | 343 | 35 | 0 | CC | AC | AG | CG | TC | CC | AA | AG | CC |
| 0682 | 15029 | 277 | 55 | 16 | CC | AA | AG | CG | TC | CC | AA | AA | CC |
| 0683 | 13151 | 254 | 94 | 41 | CC | AC | AG | CG | TC | CC | AT | AG | CG |
| 0684 | 14588 | 291 | 99 | 9 | TC | AA | AG | CC | CC | CC | AA | AA | CC |
| 0685 | 11483 | 262 | 104 | 36 | TT | AA | AA | CC | CC | TT | AA | AA | CC |
| 0686 | 15867 | 283 | 110 | 16 | TT | AC | AG | CG | TC | CC | AT | AG | GG |
| 0688 | 11086 | 271 | 101 | 25 | TC | AA | AA | CC | CC | TC | AA | AA | CC |
| 0689 | 13645 | 256 | 116 | 38 | TT | AC | AG | CG | TC | CC | AT | AG | GG |
| 0690 | 10867 | 261 | 77 | 24 | TT | AA | AG | CG | TC | CC | AA | AG | CC |
| 0692 | 11331 | 315 | 66 | 0 | CC | AC | AG | CG | TC | TC | AA | AG | CG |
| 0693 | 13558 | 270 | 129 | 31 | TT | AC | AG | CG | TC | CC | AA | AA | CC |
| 0694 | 12524 | 263 | 68 | 37 | CC | CC | GG | CC | TT | CC | AA | GG | CC |
| 0695 | 09804 | 290 | 100 | 9 | CC | AA | GG | CG | TC | CC | AA | AA | CC |
| 0696 | 15861 | 240 | 126 | 51 | CC | AC | AG | CG | TC | CC | AT | AG | CG |
| 0697 | 14704 | 261 | 100 | 32 | CC | AC | AG | CG | TC | TC | AT | AG | CG |
| 0698 | 13572 | 311 | 51 | 0 | CC | CC | GG | CC | TT | CC | AA | GG | CC |
| 0700 | 10949 | 289 | 86 | 12 | TC | AA | AA | CC | CC | TC | AA | AA | CC |
| 0701 | 11175 | 274 | 88 | 16 | CC | AA | AG | CC | CC | TC | AA | AA | CC |
| 0703 | 13474 | 240 | 59 | 36 | CC | CC | GG | CC | TT | CC | AA | GG | GG |
| 0705 | 13873 | 289 | 53 | 11 | CC | AA | AA | CC | CC | TC | AA | AA | CC |
| 0706 | 10568 | 268 | 65 | 28 | CC | AC | AG | CG | TC | CC | AA | AG | CG |
| 0710 | 11078 | 269 | 64 | 14 | CC | AA | AG | CC | CC | TC | AT | AA | CG |
| 0872 | 13866 | 285 | 67 | 15 | TT | AC | AG | CG | TC | CC | AT | AG | CC |
| 0873 | 14289 | 275 | 101 | 17 | TC | AC | AG | CG | TC | CC | AT | AG | CG |
| 0874 | 14347 | 255 | 126 | 44 | CC | AA | AG | CC | CC | TC | AA | AA | CC |
| 0875 | 12348 | 278 | 82 | 23 | CC | AC | GG | CG | TC | CC | AT | AG | CC |
| 0876 | 15447 | 247 | 83 | 40 | TC | AA | AA | CC | CC | CC | AA | AA | CC |
| 0878 | 14444 | 241 | 77 | 54 | TC | AA | AA | CC | CC | TC | AA | AA | GG |
| 0880 | 15576 | 267 | 76 | 33 | TT | CC | AA | CC | CC | CC | AA | AA | CC |
| 0881 | 10578 | 255 | 101 | 42 | TC | AA | AA | CC | CC | CC | AA | AA | GG |
| 0882 | 15653 | 298 | 54 | 3 | CC | AC | AG | CG | TC | TC | AT | AG | CG |
| 0883 | 15175 | 288 | 83 | 13 | CC | CC | GG | CC | TT | CC | AA | GG | CG |
| 0885 | 11607 | 240 | 111 | 53 | CC | AC | AG | CC | TC | TC | AT | AG | CG |
| 0886 | 14862 | 282 | 65 | 19 | CC | CC | GG | CC | TT | CC | AA | GG | CC |
| 0887 | 15617 | 275 | 103 | 23 | TT | AA | AG | CG | TC | CC | AA | AA | CC |
| 0888 | 14502 | 258 | 105 | 41 | CC | AC | AG | CG | TC | TT | AT | AG | CC |
| 0889 | 11399 | 258 | 65 | 39 | CC | AA | GG | CC | CC | CC | AA | AA | CC |
| 0890 | 14004 | 294 | 75 | 7 | CC | AA | AA | CC | CC | TC | AA | AA | CC |
| 0891 | 14654 | 274 | 65 | 25 | TT | AA | AA | CC | CC | CC | AA | AA | CC |
| 0892 | 11842 | 291 | 55 | 10 | CC | AC | AG | CG | TC | TC | AT | AG | CG |
| 0893 | 09949 | 288 | 70 | 12 | TT | AC | AG | CG | TC | CC | AT | AG | CC |
| 0894 | 13583 | 296 | 52 | 5 | TT | AC | AG | CG | TC | CC | AT | AG | CC |
| 0897 | 11874 | 253 | 82 | 32 | CC | CC | GG | CC | TT | CC | AA | GG | GG |
| 0898 | 15214 | 251 | 97 | 36 | CC | AA | AA | CC | CC | TC | AA | AA | GG |
| 0899 | 11332 | 303 | 76 | 0 | CC | AA | AA | CC | CC | TC | AA | AA | CC |
| 0900 | 12528 | 283 | 44 | 11 | CC | CC | GG | CC | TT | CC | AA | GG | CC |
| 0901 | 10720 | 284 | 65 | 7 | CC | CC | GG | CC | TT | CC | AA | GG | CC |
| 0902 | 14219 | 267 | 82 | 34 | TT | AA | AA | CC | CC | CC | AT | GG | CG |
| 0903 | 15452 | 258 | 128 | 40 | TC | AC | AG | CG | TC | CC | AT | AG | CG |
| 0904 | 15294 | 259 | 59 | 16 | TC | AA | AA | CC | CC | TC | AA | AA | CC |
| 0905 | 12623 | 268 | 50 | 30 | CC | AA | AA | CC | CC | TC | AA | AA | CC |
| 0906 | 13631 | 274 | 70 | 25 | CC | CC | GG | CC | TT | CC | AA | GG | CC |
| 0907 | 13287 | 299 | 63 | 2 | CC | AA | GG | CC | CC | CC | AA | AA | CC |
| 0908 | 13573 | 300 | 57 | 1 | CC | CC | GG | CC | TT | CC | AA | GG | CG |
| 0909 | 12558 | 276 | 80 | 23 | CC | AA | AG | CC | CC | TC | AA | AA | CC |
| 0926 | 12981 | 254 | 58 | 39 | TC | AA | AG | CC | CC | CC | AA | AA | CC |
| 0927 | 14411 | 278 | 102 | 20 | TT | AA | AA | CC | CC | CC | AA | AA | CC |
| 0928 | 15655 | 286 | 60 | 14 | TC | AA | AA | CC | CC | TC | AA | AA | CC |
| 0929 | 12361 | 307 | 32 | 0 | CC | CC | GG | CC | TT | CC | AA | GG | CG |
| 0930 | 10554 | 254 | 133 | 47 | CC | AA | AG | CC | CC | CC | AA | AA | CC |
| 0931 | 11818 | 303 | 47 | 0 | CC | AA | GG | CC | CC | CC | AA | AA | CC |
| 0932 | 10865 | 282 | 75 | 8 | TC | AA | AG | CC | CC | CC | AA | AA | CC |
| 0934 | 10952 | 286 | 85 | 11 | CC | AA | AA | GG | CC | TT | TT | AA | GG |
| 0936 | 15700 | 275 | 74 | 19 | TC | AA | AA | GG | CC | CC | TT | AA | GG |
| 0938 | 10937 | 263 | 83 | 35 | CC | AA | AA | GG | CC | TT | TT | AA | GG |
| 0940 | 15454 | 265 | 47 | 27 | TC | AA | AA | GG | CC | CC | TT | AA | GG |
| 0943 | 10306 | 297 | 50 | 3 | TT | AC | AA | CG | TC | CC | TT | AG | CG |
| 0944 | 12087 | 295 | 76 | 6 | CC | AA | AG | GG | CC | TC | TT | AA | GG |
| 0945 | 11377 | 279 | 98 | 17 | TT | AA | AA | GG | CC | CC | TT | AA | GG |
| 0947 | 12726 | 273 | 56 | 20 | TT | AA | AA | GG | CC | CC | TT | AA | GG |
| 0951 | 13216 | 269 | 102 | 27 | CC | AA | AA | GG | CC | CC | TT | AA | GG |
| 0955 | 11541 | 302 | 87 | 0 | TC | AA | AA | GG | CC | TC | TT | AA | GG |
| 0956 | 14753 | 254 | 134 | 44 | CC | AA | AA | GG | CC | TT | TT | AA | GG |
| 0958 | 12616 | 268 | 133 | 32 | TT | AA | AA | GG | CC | CC | TT | AA | GG |
| 0960 | 11964 | 273 | 52 | 25 | TT | AC | AG | CG | TC | CC | TT | AG | GG |
| 0961 | 15223 | 272 | 69 | 23 | TT | AC | AG | CG | TC | CC | TT | AG | GG |
| 0962 | 14295 | 312 | 54 | 0 | CC | AC | GG | CG | TC | CC | TT | AG | GG |
| 0963 | 09869 | 276 | 59 | 20 | CC | AA | AA | GG | CC | CC | TT | AA | GG |
| 1084 | 14065 | 285 | 81 | 11 | TT | AC | AG | CG | TC | CC | AT | AG | GG |
| 1086 | 10915 | 274 | 48 | 26 | TT | AC | AG | GG | TC | CC | AT | AG | GG |
| 1087 | 10940 | 264 | 108 | 35 | CC | AA | AA | GG | CC | TC | TT | AA | GG |
| 1088 | 11231 | 281 | 78 | 18 | TT | AA | AA | GG | CC | CC | TT | AA | GG |
| 1089 | 14823 | 276 | 78 | 21 | CC | AC | AG | CG | TC | CC | AA | AG | CG |
| 1090 | 14799 | 286 | 58 | 15 | TC | AA | AA | GG | CC | TC | TT | AA | GG |
| 1091 | 09921 | 254 | 82 | 39 | CC | AC | GG | CG | TC | CC | AA | AG | CG |
| 1094 | 13419 | 263 | 76 | 34 | CC | AA | AA | GG | CC | CC | TT | AA | GG |
| 1095 | 09834 | 252 | 133 | 34 | TC | AA | AA | GG | CC | CC | TT | AA | GG |
| 1096 | 15066 | 296 | 50 | 4 | CC | AA | AA | GG | CC | CC | TT | AA | GG |
| 1097 | 12585 | 256 | 94 | 43 | CC | AA | AG | GG | CC | TC | TT | AA | GG |
| 1102 | 13821 | 309 | 38 | 0 | TT | AC | AG | CG | TC | CC | AT | AG | CG |
| 1103 | 11578 | 267 | 75 | 30 | TC | AA | AA | GG | CC | TC | TT | AA | GG |
| 1104 | 14560 | 291 | 77 | 9 | CC | AA | AA | GG | CC | TT | TT | AA | GG |
| 1106 | 12382 | 252 | 65 | 41 | CC | CC | GG | CC | TT | CC | AA | GG | CC |
| 1107 | 09887 | 268 | 101 | 31 | CC | CC | GG | CC | TT | CC | AA | GG | GG |
| 1108 | 13344 | 283 | 64 | 5 | CC | AA | GG | GG | CC | CC | TT | AA | GG |
| 1109 | 15543 | 293 | 80 | 8 | TT | AC | AG | CG | TC | CC | AT | AG | CG |
| 1110 | 15190 | 290 | 62 | 10 | TC | AC | AG | CG | TC | CC | TT | AG | GG |
| 1111 | 11472 | 288 | 54 | 5 | CC | AC | AG | CC | TC | TC | TT | AG | GG |
| 1112 | 11772 | 264 | 69 | 32 | TC | AC | AG | CG | TC | CC | AT | AG | GG |
| 1113 | 10864 | 297 | 40 | 2 | CC | AA | GG | GG | CC | CC | TT | AG | GG |
| 1114 | 10761 | 262 | 115 | 36 | TC | AA | AA | GG | CC | TC | TT | AA | GG |
| 1116 | 13130 | 253 | 95 | 40 | CC | AA | GG | GG | CC | CC | TT | AA | GG |
| 1117 | 15212 | 273 | 120 | 27 | TC | AA | AA | GG | CC | TC | TT | AA | GG |
| 1118 | 13740 | 300 | 42 | 1 | TT | AA | AA | GG | CC | CC | TT | AA | GG |
| 1120 | 15682 | 285 | 94 | 13 | CC | CC | GG | CC | TT | CC | AT | GG | CC |
| 1121 | 10618 | 275 | 83 | 24 | CC | CC | GG | CC | TT | CC | AA | GG | CC |
| 1192 | 15761 | 297 | 36 | 4 | TT | AA | AG | CC | CC | TC | AA | AG | CC |
| 1193 | 14666 | 266 | 72 | 14 | TT | AA | AA | CC | CC | CC | AA | AG | CC |
| 1194 | 14939 | 280 | 60 | 16 | CC | CC | GG | CC | TT | CC | AA | GG | CC |
| 1195 | 09794 | 253 | 113 | 38 | CC | AC | AG | CG | TC | TC | AT | AG | CC |
| 1196 | 14412 | 286 | 77 | 12 | CC | CC | GG | CC | TT | CC | AA | GG | CG |
| 1197 | 13998 | 301 | 45 | 0 | CC | AA | AG | CC | CC | TC | AA | AA | CC |
| 1199 | 15251 | 269 | 54 | 29 | TT | AA | AA | CC | CC | CC | AA | AA | CC |
| 1200 | 13243 | 293 | 53 | 8 | TT | AC | AG | CG | TC | CC | AT | AG | CC |
| 1201 | 10757 | 271 | 67 | 26 | TC | AA | AA | CC | CC | CC | AA | AA | CC |
| 1202 | 14009 | 288 | 84 | 11 | CC | CC | GG | CC | TT | CC | AA | GG | CC |
| 1203 | 14900 | 267 | 88 | 27 | CC | CC | GG | CC | TT | CC | AA | GG | GG |
| 1205 | 09952 | 288 | 103 | 12 | TC | AA | AA | CC | CC | TC | AA | AA | CC |
| 1206 | 15350 | 267 | 46 | 20 | TC | AA | AA | CC | CC | CC | AA | AA | CC |
| 1208 | 14639 | 269 | 53 | 32 | CC | AC | AG | CG | TC | TC | AT | AG | CG |
| 1209 | 12730 | 278 | 59 | 21 | TC | AC | AG | CG | TC | CC | AT | AG | CC |
| 1210 | 10349 | 269 | 62 | 25 | TC | AC | AG | CG | TC | CC | AA | AG | CG |
| 1211 | 13265 | 263 | 108 | 30 | CC | AC | AG | CG | TC | TT | AA | AA | CC |
| 1212 | 11193 | 291 | 85 | 9 | TT | AA | AG | CG | TC | CC | AA | AA | CC |
| 1213 | 11761 | 253 | 66 | 41 | CC | AA | AG | CC | CC | CC | AA | AA | CC |
| 1214 | 12371 | 276 | 71 | 23 | CC | AA | AA | CC | CC | TT | AA | AA | CC |
| 1215 | 11081 | 273 | 107 | 27 | CC | AC | AG | CG | TC | TC | AA | AG | CC |
| 1216 | 13811 | 299 | 55 | 2 | CC | CC | GG | CC | TT | CC | AA | GG | CC |
| 1217 | 11219 | 290 | 35 | 9 | TC | AA | AG | CC | CC | CC | AA | AA | CC |
| 1218 | 12310 | 273 | 34 | 13 | CC | AC | AG | CG | TC | TC | AT | AG | CG |
| 1220 | 15758 | 254 | 82 | 44 | CC | CC | GG | CC | TT | CC | AA | GG | CG |
| 1221 | 14178 | 270 | 89 | 25 | TC | AA | AA | CC | CC | CC | AA | AA | CC |
| 1222 | 10510 | 276 | 88 | 24 | TT | AA | AA | CC | CC | CC | AA | AA | CC |
| 1223 | 12390 | 349 | 32 | 0 | TT | AA | AG | CG | TC | CC | AA | AA | CC |
| 1224 | 12005 | 300 | 33 | 1 | TC | AA | AA | CC | CC | TC | AA | AA | CC |
| 1225 | 11358 | 256 | 62 | 16 | CC | CC | GG | CC | TT | CC | AA | GG | CG |
| 1226 | 14557 | 282 | 79 | 12 | CC | AA | AA | CC | CC | TT | AA | AA | CC |
| 1227 | 13692 | 285 | 107 | 11 | CC | AA | AA | CC | CC | TC | AA | AA | CC |
| 1228 | 15604 | 255 | 96 | 40 | TC | AA | AA | CC | CC | TC | AA | AA | CC |
| 1229 | 12486 | 251 | 105 | 39 | TC | AA | AA | CC | CC | CC | AA | AA | CC |
| 1230 | 10197 | 254 | 92 | 14 | TT | AA | AG | CG | TC | TT | AA | AA | CC |
| 1231 | 14717 | 257 | 68 | 35 | CC | CC | GG | CC | TT | CC | AA | GG | CC |
| 1321 | 11249 | 264 | 69 | 29 | CC | CC | GG | CC | TT | CC | AA | GG | CC |
| 1322 | 12989 | 303 | 75 | 0 | TC | AA | AA | CC | CC | CC | AA | AA | CC |
| 1324 | 13614 | 303 | 41 | 0 | CC | CC | GG | CC | TT | CC | AA | GG | CG |
| 1325 | 14846 | 259 | 102 | 40 | CC | CC | GG | CC | TT | CC | AA | GG | CG |
| 1326 | 13197 | 280 | 76 | 15 | CC | CC | GG | CC | TT | CC | AA | GG | CG |
| 1327 | 11346 | 277 | 90 | 22 | CC | AA | AG | CC | TC | CC | AA | AA | CC |
| 1328 | 13309 | 271 | 95 | 21 | CC | AC | GG | CG | TC | CC | AA | AA | CC |
| 1329 | 12163 | 281 | 57 | 16 | CC | AC | AG | CC | TC | TC | AA | AA | CG |
| 1330 | 14535 | 286 | 71 | 7 | TC | AA | AA | CC | CC | TC | AA | AA | CC |
| 1331 | 09975 | 261 | 56 | 26 | CC | AA | AG | CC | CC | TC | AA | AA | CC |
| 1332 | 11440 | 270 | 70 | 31 | CC | CC | GG | CC | TT | CC | AA | GG | CC |
| 1333 | 11832 | 277 | 122 | 21 | CC | AC | AG | CG | TC | TC | AT | AG | CG |
| 1334 | 14343 | 298 | 51 | 3 | CC | AA | AG | CC | CC | TC | AA | AA | CC |
| 1335 | 11711 | 295 | 55 | 5 | CC | CC | GG | CC | TT | CC | AA | GG | CC |
| 1337 | 12333 | 296 | 67 | 5 | CC | AA | AG | CC | CC | CC | AA | AA | CC |
| 1338 | 12933 | 282 | 64 | 19 | CC | AC | GG | CG | TC | CC | AT | AG | CG |
| 1339 | 15487 | 273 | 102 | 26 | CC | AA | GG | CC | TC | CC | AA | AA | CC |
| 1340 | 13018 | 300 | 36 | 1 | CC | AA | AG | CC | CC | CC | AA | GG | CC |
| 1341 | 13214 | 298 | 41 | 2 | CC | CC | GG | CC | TT | CC | AA | GG | CC |
| 1342 | 13794 | 277 | 50 | 22 | CC | AC | AG | CG | TC | TC | AA | AA | CC |
| 1343 | 11931 | 264 | 51 | 33 | CC | CC | GG | CC | TT | CC | AA | GG | CC |
| 1344 | 13618 | 292 | 105 | 9 | CC | AC | AG | CG | TC | TC | AT | AG | CG |
| 1345 | 15381 | 253 | 76 | 17 | CC | AC | AG | CG | TC | CC | AT | AG | CG |
| 1346 | 12907 | 240 | 101 | 43 | TT | AA | AG | CG | TC | CC | AA | AG | GG |
| 1347 | 10401 | 262 | 44 | 10 | CC | CC | GG | CC | TT | CC | AA | AG | CC |
| 1348 | 13200 | 269 | 81 | 24 | TC | AA | AA | CC | CC | TC | AA | AA | CC |
| 1349 | 12179 | 263 | 76 | 8 | CC | AC | AG | CG | TC | CC | AT | AG | CG |
| 1350 | 12617 | 253 | 126 | 42 | TC | AC | AG | CG | TC | CC | AT | AG | GG |
| 1351 | 11183 | 301 | 37 | 0 | CC | AA | GG | CG | CC | CC | AA | AA | CC |
| 1352 | 13870 | 287 | 104 | 10 | TC | AA | AA | GG | CC | CC | TT | AA | GG |
| 1353 | 15352 | 288 | 64 | 8 | TC | AA | AA | GG | CC | CC | TT | AA | GG |
| 1355 | 15433 | 252 | 57 | 44 | CC | CC | GG | CC | TT | CC | AA | GG | GG |
| 1357 | 13889 | 267 | 86 | 32 | CC | AA | AA | GG | CC | TT | TT | AA | GG |
| 1358 | 13867 | 294 | 62 | 6 | TT | AA | AG | CG | TC | CC | TT | AG | GG |
| 1359 | 14357 | 292 | 50 | 9 | CC | AA | GG | CG | TC | CC | TT | AG | CG |
| 1361 | 11670 | 269 | 123 | 29 | CC | AA | GG | GG | CC | CC | TT | AA | GG |
| 1363 | 11251 | 263 | 42 | 28 | CC | AA | AA | GG | CC | CC | TT | AA | GG |
| 1364 | 12105 | 280 | 80 | 16 | TT | AA | AA | GG | CC | CC | TT | AA | GG |
| 1453 | 09914 | 273 | 67 | 27 | CC | CC | GG | CC | TT | CC | AA | GG | GG |
| 1454 | 12519 | 263 | 133 | 36 | TT | AA | AA | GG | CC | CC | TT | AA | GG |
| 1455 | 10095 | 265 | 120 | 32 | CC | AA | AA | GG | CC | CC | TT | AA | GG |
| 1456 | 13329 | 285 | 49 | 11 | TT | AA | AA | CG | TC | CC | AT | AG | GG |
| 1457 | 11804 | 271 | 78 | 28 | CC | CC | GG | CC | TT | CC | AA | GG | CC |
| 1458 | 13361 | 255 | 77 | 40 | TC | AA | AA | GG | CC | TC | TT | AA | GG |
| 1462 | 10021 | 303 | 32 | 0 | CC | CC | GG | CG | TC | CC | AA | GG | GG |
| 1463 | 13068 | 279 | 58 | 13 | CC | CC | GG | CC | TT | CC | AA | GG | GG |
| 1464 | 13874 | 279 | 70 | 18 | TT | AA | AA | GG | CC | CC | TT | AA | GG |
| 1465 | 15690 | 277 | 68 | 18 | TC | AA | AG | GG | CC | CC | TT | AA | GG |
| 1466 | 15210 | 249 | 114 | 47 | CC | AA | AA | GG | CC | TC | TT | AA | GG |
| 1467 | 15250 | 297 | 89 | 4 | TT | AA | AA | GG | CC | CC | TT | AA | GG |
| 1468 | 11184 | 276 | 79 | 21 | CC | AA | GG | GG | CC | CC | TT | AA | GG |
| 1469 | 15654 | 279 | 92 | 20 | TT | AA | AA | GG | CC | CC | TT | AA | GG |
| 1470 | 11265 | 279 | 66 | 16 | CC | AA | AA | GG | CC | CC | TT | AA | GG |
| 1472 | 10747 | 247 | 93 | 47 | CC | AC | AG | CG | TC | TC | AT | AG | CG |
| 1475 | 15891 | 304 | 38 | 0 | CC | AA | AA | GG | CC | CC | TT | AA | GG |
| 1476 | 10776 | 316 | 81 | 0 | CC | AC | AG | CG | TC | TC | AT | AG | CG |
| 1477 | 11532 | 288 | 100 | 9 | CC | CC | GG | CC | TT | CC | AA | GG | GG |
| 1478 | 15399 | 257 | 76 | 42 | CC | AA | GG | GG | CC | CC | TT | AA | GG |
| 1479 | 12565 | 277 | 89 | 16 | CC | AA | AA | GG | CC | TT | TT | AA | GG |
| 1481 | 12274 | 277 | 59 | 23 | TT | AA | AA | GG | CC | CC | TT | AA | GG |
| 1483 | 11344 | 285 | 59 | 15 | CC | AC | AG | CG | TC | CC | AT | AG | CG |
| 1484 | 15883 | 277 | 67 | 15 | TT | AC | AG | CG | TC | CC | AT | AG | CG |
| 1485 | 10304 | 312 | 58 | 0 | TT | AA | AA | GG | CC | CC | TT | AA | GG |
| 1486 | 12177 | 269 | 98 | 31 | CC | CC | GG | CC | TT | CC | AA | GG | GG |
| 1488 | 13371 | 266 | 80 | 31 | TC | AA | AA | GG | CC | CC | TT | AA | GG |
| 1489 | 14067 | 250 | 60 | 23 | CC | AA | AG | GG | CC | TC | TT | AA | GG |
| 1490 | 13115 | 250 | 110 | 21 | TT | AA | AA | GG | CC | CC | TT | AA | GG |
| 1492 | 14708 | 250 | 139 | 49 | TC | AA | AA | GG | CC | TC | TT | AA | GG |
| 1493 | 11682 | 285 | 54 | 12 | TT | AA | AG | CG | TC | CC | AT | AG | GG |
| 1494 | 12028 | 270 | 82 | 28 | CC | AA | GG | GG | CC | CC | TT | AA | GG |
| 1495 | 12768 | 253 | 120 | 45 | TT | AA | AA | GG | CC | CC | TT | AA | GG |
| 1606 | 17249 | 280 | 37 | 15 | CC | CC | GG | CC | TT | CC | AA | GG | CC |
| 1609 | 16806 | 308 | 74 | 0 | CC | AA | AA | CG | TC | CC | AT | AG | GG |
| 1611 | 21905 | 282 | 82 | 18 | TT | AC | AG | CG | TT | CC | AT | AG | CG |
| 1612 | 18309 | 299 | 88 | 1 | CC | AC | GG | CG | TC | CC | AT | AG | CG |
| 1616 | 19323 | 255 | 88 | 38 | CC | AA | GG | GG | TC | CC | TT | AG | GG |
| 1618 | 21451 | 292 | 57 | 7 | CC | CC | GG | CC | TT | CC | AA | GG | CG |
| 1621 | 21196 | 270 | 52 | 18 | TC | AC | AG | CG | TC | CC | AT | AG | CG |
| 1622 | 20357 | 270 | 92 | 28 | CC | CC | GG | CC | TT | CC | AA | GG | CG |
| 1623 | 18986 | 268 | 45 | 23 | TC | AA | AA | GG | CC | TC | TT | AA | GG |
| 1624 | 19453 | 305 | 30 | 0 | CC | CC | GG | CC | TT | CC | AA | GG | GG |
| 1626 | 16097 | 284 | 43 | 15 | TT | AC | AG | CG | TC | CC | AT | AG | CG |
| 1628 | 21502 | 278 | 59 | 19 | CC | AA | AA | GG | CC | TC | TT | AA | GG |
| 1629 | 17856 | 290 | 40 | 8 | CC | CC | GG | CC | TT | CC | AA | GG | CC |
| 1630 | 20857 | 271 | 56 | 25 | TC | AA | AG | GG | CC | CC | TT | AA | GG |
| 1631 | 20309 | 288 | 32 | 10 | CC | AC | AA | CG | TC | TC | AT | AG | CG |
| 1632 | 21797 | 272 | 76 | 23 | TC | AA | AG | GG | CC | CC | TT | AA | GG |
| 1635 | 16137 | 285 | 54 | 13 | TC | AA | AA | GG | CC | TC | TT | AA | GG |
| 1638 | 17741 | 302 | 36 | 0 | TT | AC | AG | CG | TC | CC | AT | AG | CG |
| 1639 | 19983 | 299 | 65 | 1 | TT | AC | AG | CG | TC | CC | AT | AG | CG |
| 1640 | 19582 | 336 | 56 | 0 | CC | CC | GG | CC | TT | CC | AA | GG | GG |
| 1642 | 15967 | 288 | 88 | 10 | CC | AC | AG | CG | TC | TC | AT | AG | GG |
| 1643 | 20262 | 297 | 76 | 2 | CC | AA | AA | GG | CC | TT | TT | AA | GG |
| 1647 | 21330 | 280 | 30 | 2 | CC | CC | GG | CC | TT | CC | AA | GG | GG |
| 1648 | 20461 | 280 | 58 | 1 | TT | AA | AA | GG | CC | CC | TT | AA | GG |
| 1649 | 20282 | 280 | 70 | 15 | TC | AA | AA | GG | CC | TC | TT | AA | GG |
| 1653 | 16300 | 270 | 60 | 24 | CC | AA | AA | GG | CC | TT | TT | AA | GG |
| 1655 | 18693 | 280 | 44 | 1 | CC | AA | GG | GG | CC | CC | TT | AA | GG |
| 1656 | 21292 | 280 | 79 | 2 | CC | AC | AG | CG | TC | CC | AT | AG | CG |
| 1657 | 20459 | 280 | 30 | 1 | TT | AC | AA | CG | TC | CC | TT | AG | GG |
| 1663 | 18064 | 272 | 92 | 26 | TT | AC | AG | CG | TC | CC | AT | AG | GG |
| 1665 | 19100 | 289 | 43 | 10 | CC | CC | GG | CC | TT | CC | AA | GG | GG |
| 1734 | 21638 | 284 | 92 | 12 | TC | AA | AG | GG | CC | CC | TT | AA | GG |
| 1736 | 18744 | 245 | 66 | 47 | CC | AA | GG | GG | CC | CC | TT | AA | GG |
| 1738 | 17414 | 272 | 73 | 22 | CC | AA | GG | GG | CC | CC | TT | AA | GG |
| 1740 | 17665 | 271 | 45 | 15 | CC | CC | GG | CC | TT | CC | AA | GG | GG |
| 1745 | 18399 | 279 | 110 | 18 | CC | AA | GG | CG | TC | CC | AT | AG | GG |
| 1749 | 19722 | 284 | 48 | 13 | CC | AA | GG | GG | CC | CC | TT | AG | GG |
| 1750 | 16926 | 289 | 84 | 11 | CC | AC | AG | CG | TC | CC | AT | AG | GG |
| 1751 | 17597 | 278 | 49 | 20 | TC | AA | AA | GG | CC | CC | TT | AA | GG |
| 1754 | 21426 | 338 | 40 | 0 | CC | AA | GG | CG | TC | CC | AT | AG | GG |
| 1756 | 19287 | 288 | 36 | 9 | TT | AC | AG | CG | TC | CC | AT | AG | GG |
| 1758 | 16798 | 282 | 67 | 14 | TC | AA | AA | GG | CC | CC | TT | AA | GG |
| 1762 | 21197 | 252 | 80 | 29 | TC | AC | AG | CG | TC | TT | AT | AG | CG |
| 1767 | 17284 | 282 | 52 | 13 | CC | AC | AG | CG | TC | CC | AT | AG | GG |
| 1769 | 18785 | 341 | 33 | 0 | TT | AA | AA | GG | CC | CC | TT | GG | GG |
| 1771 | 18426 | 300 | 70 | 0 | CC | CC | GG | CC | TT | CC | AA | GG | CG |
| 1772 | 21821 | 335 | 36 | 0 | CC | AA | AA | GG | CC | TC | TT | AA | GG |
| 1775 | 16938 | 258 | 40 | 32 | CC | CC | GG | CC | TT | CC | AA | GG | CG |
| 1779 | 17866 | 282 | 80 | 18 | TT | AA | AA | GG | CC | CC | TT | AG | CG |
| 1780 | 17380 | 287 | 39 | 13 | CC | CC | GG | CC | TT | CC | AA | GG | GG |
| 1781 | 19873 | 280 | 38 | 16 | TC | AA | AA | GG | CC | CC | TT | AA | GG |
| 1782 | 18260 | 285 | 78 | 14 | CC | AC | GG | CG | TC | CC | AT | AG | CG |
| 1786 | 17404 | 296 | 66 | 4 | CC | AA | AG | GG | CC | CC | TT | AA | GG |
| 1787 | 20910 | 270 | 37 | 26 | TC | AA | AA | GG | CC | TC | TT | AA | GG |
| 1789 | 18328 | 280 | 31 | 15 | CC | AA | AA | GG | CC | TT | TT | AA | GG |
| 1790 | 21909 | 288 | 91 | 12 | CC | AA | GG | GG | TC | CC | TT | AA | GG |
| 1791 | 20253 | 320 | 59 | 0 | CC | CC | GG | CC | TT | CC | AA | GG | CG |
| 1792 | 18752 | 292 | 68 | 8 | CC | AA | AA | GG | CC | CC | TT | AA | GG |
| 1794 | 16913 | 301 | 66 | 0 | CC | CC | GG | CC | TT | CC | AA | GG | CC |
| 1797 | 19571 | 286 | 47 | 14 | TT | AC | AG | CG | TC | CC | AT | AG | GG |
| 1798 | 19496 | 293 | 37 | 7 | CC | AA | GG | GG | TC | CC | TT | AG | GG |
| 1801 | 17736 | 252 | 115 | 45 | CC | CC | GG | CC | TT | CC | AA | GG | CC |
| 1802 | 20540 | 258 | 84 | 35 | TT | AC | AG | CG | TC | CC | TT | AG | CG |
| 1804 | 17447 | 269 | 78 | 30 | TC | AA | AG | GG | CC | CC | TT | AA | GG |
| 1809 | 17833 | 252 | 84 | 48 | TC | AA | AA | GG | CC | CC | TT | AA | GG |
| 1812 | 20147 | 270 | 108 | 26 | CC | AA | GG | GG | CC | CC | TT | AA | GG |
| 1813 | 21783 | 270 | 91 | 28 | CC | AA | AA | GG | CC | CC | TT | AA | GG |
| 1818 | 22047 | 253 | 132 | 42 | TT | AC | AG | CG | TC | TT | AT | AG | CG |
| 1820 | 16689 | 268 | 66 | 27 | TT | AC | AG | CG | TC | CC | AT | AG | GG |
| 1822 | 20896 | 271 | 104 | 29 | TC | AA | AA | GG | CC | TC | TT | AA | GG |
| 1825 | 19591 | 263 | 77 | 36 | CC | CC | GG | CC | TT | CC | AA | GG | CG |
| 1827 | 18257 | 261 | 89 | 34 | CC | CC | GG | CC | TT | CC | AA | GG | GG |
| 1828 | 20352 | 271 | 86 | 28 | CC | CC | GG | CC | TT | CC | AA | GG | CG |
| 1832 | 21552 | 248 | 80 | 49 | TC | AA | AA | GG | CC | CC | TT | AA | GG |
| 1835 | 17602 | 278 | 61 | 22 | CC | AA | AA | GG | CC | TT | TT | AG | GG |
| 1838 | 22002 | 273 | 102 | 26 | CC | AA | AA | GG | CC | CC | TT | AA | GG |
| 1840 | 20708 | 248 | 111 | 50 | CC | CC | GG | CC | CC | CC | AA | GG | CG |
| 1845 | 18908 | 267 | 67 | 30 | TT | AA | AA | GG | CC | CC | TT | AG | GG |
| 1847 | 21356 | 271 | 109 | 29 | CC | AA | AA | GG | TC | TC | TT | AG | GG |
| 1849 | 20829 | 262 | 62 | 37 | CC | AC | AG | GG | TC | TC | AT | AG | CG |
| 1852 | 18980 | 267 | 77 | 31 | CC | CC | GG | CC | TT | CC | AA | GG | CG |
| 1854 | 18593 | 272 | 71 | 27 | CC | AC | GG | GG | TC | CC | TT | AG | GG |
| 1855 | 19498 | 261 | 76 | 33 | CC | AA | AG | GG | CC | CC | TT | AA | GG |
| 1857 | 19529 | 254 | 115 | 43 | CC | AC | AG | CG | TC | TC | AT | AG | CG |
| 1859 | 19482 | 267 | 73 | 29 | CC | AA | AA | GG | CC | TT | TT | AA | GG |
| 1861 | 20947 | 249 | 115 | 46 | TC | AA | AG | GG | CC | CC | TT | AA | GG |
| 1862 | 17429 | 243 | 116 | 57 | CC | AA | AA | CG | CC | CC | TT | AA | GG |
| 1863 | 21611 | 258 | 96 | 37 | CC | AA | AA | GG | CC | CC | TT | AA | GG |
| 1873 | 20751 | 246 | 124 | 53 | TT | AA | GG | GG | CC | CC | TT | AA | GG |
| 1874 | 19296 | 257 | 63 | 39 | TC | AA | AG | GG | CC | CC | TT | AA | GG |
| 1876 | 21105 | 267 | 78 | 30 | CC | AA | AG | CG | TC | CC | AT | AG | CG |
| 1878 | 21179 | 267 | 60 | 29 | CC | CC | GG | CC | TT | CC | AA | GG | CC |
| 1882 | 18905 | 270 | 95 | 26 | TC | AC | AG | CG | TC | CC | AT | AG | GG |
| 1884 | 17658 | 255 | 105 | 43 | TT | AA | AA | GG | CC | CC | TT | AA | GG |
| 2007 | 21038 | 286 | 68 | 12 | TC | AA | AA | GG | CC | TC | TT | AA | GG |
| 2011 | 17812 | 279 | 85 | 18 | TC | AA | AA | GG | CC | TC | TT | AA | GG |
| 2012 | 18859 | 275 | 44 | 20 | CC | AA | AA | GG | CC | TC | TT | AA | GG |
| 2013 | 20258 | 288 | 72 | 11 | TC | AA | AG | GG | CC | CC | TT | AG | GG |
| 2015 | 17711 | 278 | 86 | 19 | TC | AA | AG | GG | CC | TC | TT | AA | GG |
| 2016 | 19021 | 285 | 74 | 13 | TC | AA | AG | CG | CC | TC | TT | AG | GG |
| 2017 | 19054 | 271 | 46 | 26 | CC | AC | GG | CC | TC | TC | AA | AG | GG |
| 2018 | 18690 | 263 | 37 | 31 | CC | AA | AG | CC | CC | TC | TT | AG | GG |
| 2020 | 18573 | 280 | 69 | 16 | CC | CC | GG | CC | TT | CC | AA | GG | CC |
| 2021 | 19445 | 284 | 36 | 13 | CC | CC | GG | CC | TT | CC | AA | GG | CG |
| 2024 | 20313 | 288 | 45 | 9 | CC | CC | GG | CC | TT | CC | AA | GG | CC |
| 2025 | 21506 | 280 | 31 | 15 | TC | AA | AA | GG | CC | TC | TT | AA | GG |
| 2027 | 18692 | 291 | 32 | 9 | TT | AA | AA | GG | CC | CC | TT | AA | GG |
| 2028 | 21713 | 312 | 31 | 0 | CC | CC | GG | CC | TT | CC | AA | GG | GG |
| 2029 | 17016 | 274 | 39 | 24 | TC | AA | AA | GG | CC | CC | TT | AA | GG |
| 2031 | 19742 | 270 | 72 | 30 | TC | AC | AG | GG | TC | CC | TT | AG | GG |
| 2033 | 20922 | 276 | 56 | 17 | CC | AC | AG | CG | TC | CC | AA | AG | GG |
| 2036 | 20279 | 283 | 93 | 15 | TC | AC | AG | CG | TC | CC | AT | AG | CG |
| 2040 | 19553 | 303 | 50 | 0 | TC | AA | AA | GG | CC | TC | TT | AA | GG |
| 2044 | 16299 | 289 | 36 | 9 | TC | AA | AA | GG | CC | TT | TT | AA | GG |
| 2045 | 16890 | 269 | 78 | 25 | CC | CC | GG | CC | TT | CC | AA | GG | CG |
| 2048 | 19414 | 269 | 38 | 23 | CC | CC | GG | CC | TT | CC | AA | GG | CC |
| 2049 | 20421 | 293 | 50 | 7 | CC | CC | GG | CC | TT | CC | AA | GG | CG |
| 2053 | 17709 | 256 | 45 | 1 | CC | AA | AA | GG | CC | CC | TT | AA | GG |
| 2054 | 19049 | 286 | 34 | 14 | CC | CC | GG | CC | TT | CC | AA | GG | GG |
| 2058 | 19863 | 263 | 92 | 37 | CC | AA | AA | GG | CC | TC | TT | AA | GG |
| 2141 | 19611 | 241 | 40 | 40 | CC | AA | AG | CC | CC | CC | AA | AA | CC |
| 2143 | 20102 | 275 | 97 | 17 | TT | AA | AA | CC | CC | CC | AA | AA | CC |
| 2145 | 16094 | 275 | 53 | 22 | CC | CC | GG | CC | TT | CC | AA | GG | CG |
| 2146 | 20864 | 275 | 80 | 19 | CC | AA | AA | CC | CC | TT | AA | AA | CC |
| 2152 | 19334 | 281 | 69 | 19 | CC | AA | GG | CC | CC | CC | AA | AA | CC |
| 2156 | 20181 | 301 | 70 | 0 | TC | AA | AG | CG | TC | CC | AA | AG | CG |
| 2158 | 19501 | 277 | 42 | 22 | TC | AA | AA | CC | CC | TC | AA | AA | CC |
| 2159 | 16493 | 301 | 41 | 0 | CC | AC | AG | CG | TC | CC | AT | AG | CC |
| 2160 | 18953 | 285 | 44 | 15 | CC | CC | GG | CC | TT | CC | AA | GG | CG |
| 2164 | 20604 | 271 | 47 | 29 | CC | AA | AG | CC | CC | CC | AA | AA | CC |
| 2169 | 17620 | 268 | 33 | 32 | CC | AA | AG | CC | TC | CC | AA | AA | CC |
| 2170 | 19472 | 290 | 57 | 10 | CC | CC | GG | CC | TT | CC | AA | GG | CC |
| 2172 | 19890 | 305 | 57 | 0 | CC | AA | AA | CG | CC | TC | AA | AA | CC |
| 2174 | 19794 | 298 | 59 | 2 | CC | CC | GG | CC | TT | CC | AA | GG | CG |
| 2176 | 17807 | 336 | 35 | 0 | TC | AC | AG | CG | TC | CC | AT | AG | CC |
| 2178 | 16117 | 317 | 44 | 0 | CC | AA | AA | CC | CC | TT | AA | AG | CC |
| 2183 | 21236 | 283 | 56 | 15 | CC | CC | GG | CC | TT | CC | AA | GG | CG |
| 2186 | 21262 | 259 | 56 | 30 | CC | AC | AG | CG | TC | TC | AA | AA | CG |
| 2189 | 17676 | 300 | 60 | 0 | CC | AA | AG | CC | CC | CC | AA | AA | CC |
| 2190 | 19719 | 284 | 43 | 14 | TT | AA | AA | CC | CC | CC | AA | AA | CC |
| 2193 | 17102 | 301 | 61 | 0 | TT | AA | AG | CG | TC | CC | AT | AG | CC |
| 2195 | 17056 | 274 | 37 | 3 | TT | AA | AA | CC | CC | CC | AA | AA | CC |
| 2196 | 17877 | 274 | 68 | 2 | CC | AC | AG | CG | CC | TC | AA | AG | CG |
| 2200 | 22023 | 294 | 40 | 5 | CC | AC | GG | CG | TC | CC | AT | AG | CC |
| 2308 | 17592 | 237 | 153 | 62 | TT | AA | AA | GG | CC | TT | TT | AA | GG |
| 2309 | 21839 | 270 | 68 | 27 | TC | AA | AA | GG | CC | CC | TT | AA | GG |
| 2311 | 17735 | 263 | 102 | 34 | CC | CC | GG | CC | TT | CC | AA | GG | CC |
| 2312 | 21582 | 244 | 96 | 53 | CC | CC | GG | CC | TT | CC | AA | GG | CC |
| 2314 | 21224 | 270 | 111 | 27 | CC | CC | GG | CC | TT | CC | AA | AA | CC |
| 2321 | 19365 | 247 | 105 | 51 | TT | AC | AG | CG | TC | TT | AT | AG | CG |
| 2323 | 21138 | 258 | 53 | 35 | TT | AA | AG | CG | TC | CC | AT | AG | CG |
| 2328 | 19811 | 264 | 111 | 30 | CC | AA | GG | CG | TC | CC | TT | AG | CG |
| 2330 | 20854 | 262 | 135 | 35 | CC | AA | AA | GG | CC | TC | TT | AA | GG |
| 2332 | 20239 | 274 | 81 | 21 | CC | AC | AG | CG | TC | TC | AT | AG | CG |
| 2332 | 20239 | 274 | 81 | 21 | CC | CC | GG | CC | TT | CC | AA | GG | CG |
| 2334 | 17010 | 278 | 33 | 14 | CC | CC | GG | CC | TT | CC | AA | GG | CC |
| 2335 | 20901 | 276 | 51 | 15 | CC | AA | AA | CC | CC | TC | AA | AA | CC |
| 2336 | 19437 | 293 | 69 | 6 | CC | AC | AG | CG | TC | CC | AT | AG | CC |
| 2337 | 18782 | 301 | 84 | 0 | TC | AA | AA | CC | CC | CC | AA | AA | CC |
| 2338 | 16783 | 297 | 38 | 2 | CC | AA | AA | CC | CC | CC | AA | AA | CC |
| 2339 | 20014 | 270 | 111 | 28 | CC | AA | AA | GG | CC | TT | TT | AA | GG |
| 2340 | 20456 | 269 | 89 | 30 | CC | CC | GG | CC | TT | CC | AA | GG | CC |
| 2341 | 19080 | 258 | 82 | 32 | TT | AA | AA | GG | CC | CC | TT | AA | GG |
| 2342 | 18320 | 284 | 44 | 13 | CC | AC | GG | CG | TC | CC | AT | AG | CG |
| 2344 | 19275 | 271 | 39 | 19 | CC | CC | GG | CC | TT | CC | AA | GG | CC |
| 2345 | 17677 | 279 | 78 | 21 | CC | AA | AG | CC | CC | CC | AA | AA | CG |
| 2346 | 16805 | 280 | 108 | 16 | TC | AA | AA | CC | CC | CC | AA | AA | CC |
| 2347 | 19855 | 265 | 65 | 30 | CC | AC | AG | CG | TC | TC | AA | AA | CG |
| 2349 | 21617 | 291 | 67 | 9 | CC | AC | AG | CG | TC | TC | AT | AG | CG |
| 2352 | 19972 | 278 | 38 | 17 | CC | CC | GG | CC | TT | CC | AA | GG | CC |
| 2353 | 17087 | 335 | 51 | 0 | CC | AA | AG | CG | TC | CC | AA | AG | CC |
| 2355 | 19084 | 268 | 67 | 20 | TT | AA | AA | CC | CC | CC | AA | AA | CC |
| 2356 | 18120 | 335 | 38 | 0 | CC | CC | GG | CC | TT | CC | AA | GG | CC |
| 2357 | 20032 | 292 | 77 | 8 | CC | CC | GG | CC | TT | CC | AA | GG | CG |
| 2358 | 21653 | 239 | 80 | 47 | CC | AA | AA | GG | CC | TC | TT | AA | GG |
| 2359 | 21001 | 270 | 104 | 25 | TC | AA | AA | GG | CC | TC | TT | AA | GG |
| 2360 | 20828 | 282 | 103 | 19 | TC | AA | AG | CC | CC | CC | AA | AA | CC |
| 2361 | 16125 | 269 | 60 | 29 | CC | AA | AG | GG | CC | TC | TT | AA | GG |
| 2363 | 21171 | 249 | 132 | 51 | CC | AC | AG | CG | TC | TC | TT | AG | CG |
| 2364 | 18087 | 283 | 72 | 10 | CC | AC | AG | CG | TC | TC | AT | AG | CG |
| 2365 | 20093 | 258 | 43 | 24 | CC | AC | AG | CG | TC | TC | AA | AG | CC |
| 2366 | 17855 | 286 | 52 | 11 | CC | AC | AG | CC | TC | TC | AT | AG | CG |
| 2368 | 19541 | 317 | 69 | 0 | CC | CC | GG | CC | TT | CC | AA | GG | CC |
| 2369 | 21483 | 265 | 34 | 23 | CC | AC | AG | CG | TC | CC | AT | AG | CG |
| 2370 | 19783 | 256 | 135 | 42 | TC | AA | AA | GG | CC | TC | TT | AA | GG |
| 2371 | 20716 | 243 | 120 | 55 | CC | CC | AA | CC | CC | CC | AA | AA | CC |
| 2372 | 16487 | 245 | 148 | 49 | TT | AA | AG | GG | CC | CC | TT | AA | GG |
| 2373 | 21850 | 261 | 121 | 31 | CC | AA | AA | GG | CC | CC | TT | AA | GG |
| 2375 | 17285 | 257 | 89 | 32 | CC | AC | AG | CG | TC | CC | AT | AG | CG |
| 2376 | 21364 | 278 | 51 | 18 | TC | AA | AA | CC | CC | TC | AA | AA | CC |
| 2377 | 20946 | 248 | 120 | 48 | TT | AC | AG | CG | TC | CC | AT | AG | CG |
| 2378 | 16775 | 272 | 88 | 19 | CC | AA | AA | CC | CC | TC | AA | AA | CC |
| 2379 | 20717 | 259 | 91 | 35 | CC | AC | AG | CC | TC | TC | AT | AG | GG |
| 2380 | 22042 | 262 | 99 | 32 | CC | CC | GG | CC | TT | CC | AA | AA | GG |
| 2382 | 18360 | 278 | 52 | 21 | TC | AA | AA | CC | CC | TC | AA | AA | CC |
| 2385 | 20440 | 257 | 132 | 42 | CC | AC | AG | CG | TC | TC | AT | AG | GG |
| 2386 | 21556 | 257 | 65 | 34 | TT | AA | AA | GG | CC | CC | TT | AA | GG |
| 2387 | 17194 | 267 | 74 | 23 | TC | AA | AA | CC | CC | TC | AA | AA | CC |
| 2388 | 21333 | 249 | 90 | 48 | CC | CC | GG | CC | TT | CC | AA | GG | GG |
| 2389 | 19709 | 290 | 64 | 9 | CC | AC | AG | CG | TC | TC | AT | AG | CC |
| 2390 | 17217 | 274 | 68 | 10 | TC | AA | AG | CC | CC | CC | AA | AA | CC |
| 2391 | 19356 | 254 | 137 | 41 | CC | AC | GG | CG | CC | CC | AT | AG | GG |
| 2392 | 20815 | 276 | 62 | 17 | CC | AA | AA | CC | CC | TT | AA | AA | CC |
| 2394 | 21733 | 244 | 144 | 55 | TT | AA | AA | GG | CC | CC | TT | AA | GG |
| 2401 | 18734 | 265 | 90 | 29 | CC | AA | AG | GG | CC | TC | TT | AA | GG |
| 2402 | 17864 | 286 | 62 | 13 | CC | AA | GG | CC | CC | CC | AA | AA | CC |
| 2403 | 16460 | 268 | 96 | 31 | TT | AA | AA | GG | CC | CC | TT | AA | GG |
| 2404 | 16991 | 285 | 98 | 11 | TC | AA | AA | CC | CC | TC | AA | AA | CC |
| 2406 | 17698 | 269 | 120 | 27 | CC | AC | AG | CG | TC | CC | AT | AG | CG |
| 2407 | 16131 | 294 | 42 | 5 | TC | AA | AA | CC | CC | TC | AA | AA | CC |
| 2408 | 18397 | 262 | 66 | 32 | CC | CC | GG | CC | TT | CC | AA | GG | CC |
| 2409 | 16816 | 267 | 33 | 33 | CC | AA | AA | CC | CC | CC | AA | AA | CC |
| 2410 | 18539 | 267 | 88 | 32 | TT | AC | AG | CG | TC | CC | AT | AG | CG |
| 2411 | 16511 | 320 | 46 | 0 | CC | CC | GG | CC | TT | CC | AA | GG | CG |
| 2412 | 20957 | 285 | 94 | 11 | CC | CC | GG | CC | TT | CC | AA | GG | CC |
| 2413 | 18900 | 288 | 50 | 9 | TC | AA | AG | CC | CC | CC | AA | AA | CC |
| 2414 | 19874 | 288 | 36 | 10 | TC | AC | AG | CG | TC | CC | AT | AG | CG |
| 2419 | 20467 | 290 | 76 | 10 | TT | AA | AG | CG | CC | CC | AA | AA | CC |
| 2426 | 21360 | 261 | 37 | 31 | CC | AA | AG | CC | CC | TC | AA | AA | CC |
| 2427 | 18956 | 304 | 45 | 0 | CC | CC | GG | CC | TT | CC | AA | GG | CG |
| 2431 | 16973 | 285 | 63 | 14 | TC | AA | AA | CC | CC | TC | AA | AA | CC |
| 2433 | 21418 | 287 | 45 | 11 | TT | AC | AG | CG | TC | CC | AA | AG | CG |
| 2435 | 16947 | 336 | 41 | 0 | CC | AC | AG | CG | TC | CC | AT | AG | CG |
| 2436 | 22057 | 271 | 58 | 18 | CC | CC | GG | CC | TT | CC | AA | GG | CC |
| 2439 | 18046 | 284 | 90 | 15 | CC | AA | AG | CC | CC | TC | AA | AA | CC |
| 2440 | 19826 | 287 | 86 | 13 | CC | AA | AG | CC | CC | TT | AA | AA | CC |
| 2443 | 19751 | 283 | 33 | 15 | CC | AA | AA | CC | CC | TT | AA | AA | CC |
| 2445 | 20247 | 288 | 107 | 10 | CC | CC | GG | CC | TT | CC | AA | GG | CC |
| 2446 | 17057 | 284 | 74 | 13 | TT | AA | AA | CC | CC | CC | AA | AA | CC |
| 2451 | 18983 | 290 | 66 | 10 | CC | CC | GG | CC | TT | CC | AA | GG | CC |
| 2452 | 20995 | 255 | 54 | 45 | CC | AC | AG | CG | TC | TT | AA | AG | CC |
| 2454 | 19108 | 284 | 65 | 15 | CC | AA | GG | CG | TC | CC | AA | AA | CC |
| 2458 | 18334 | 257 | 31 | 31 | TC | AC | AG | CG | TC | CC | AT | AG | CC |
| 2459 | 16533 | 335 | 61 | 0 | CC | CC | GG | CC | TT | CC | AA | GG | CC |
| 2463 | 17883 | 286 | 82 | 14 | CC | AA | AG | CC | CC | TC | AA | AA | CC |
| 2464 | 21186 | 290 | 52 | 9 | TC | AC | AG | CG | TC | CC | AT | AG | CC |
| 2465 | 17743 | 266 | 36 | 19 | TC | AC | AG | CG | TC | CC | AA | AG | CG |
| 2468 | 17165 | 291 | 65 | 9 | CC | AC | AG | CG | TC | TC | AT | AG | CC |
| 2470 | 16504 | 296 | 42 | 4 | TT | AA | AA | CC | CC | CC | AA | AA | CC |
| 2529 | 19483 | 294 | 39 | 5 | CC | AC | GG | CG | TC | CC | AA | AA | CG |
| 2533 | 20625 | 309 | 77 | 0 | CC | CC | GG | CC | TT | CC | AA | GG | CC |
| 2538 | 18653 | 262 | 66 | 30 | TC | AA | AA | CC | CC | TC | AA | AA | CC |
| 2542 | 16641 | 278 | 76 | 19 | CC | CC | GG | CC | TT | CC | AA | GG | CC |
| 2543 | 19029 | 302 | 34 | 0 | TC | AC | AG | CG | TC | CC | AT | AG | CG |
| 2547 | 20951 | 251 | 88 | 38 | TT | AA | AA | CC | CC | CC | AA | AA | CC |
| 2552 | 19530 | 251 | 41 | 34 | CC | AC | AG | CG | TC | TC | AT | AG | CG |
| 2556 | 22113 | 270 | 80 | 25 | CC | AA | GG | CG | CC | CC | AA | AA | CC |
| 2557 | 18127 | 285 | 59 | 15 | CC | CC | GG | CC | TT | CC | AA | GG | CC |
| 2560 | 20105 | 295 | 47 | 4 | TC | AA | AA | CC | CC | TC | AA | AA | CC |
| 2562 | 21471 | 291 | 79 | 9 | CC | AA | AG | CG | CC | TT | AA | AA | CC |
| 2563 | 18103 | 285 | 46 | 14 | CC | AC | AG | CG | TC | CC | AT | AG | CG |
| 2566 | 16655 | 288 | 36 | 11 | CC | AA | GG | CG | CC | CC | AA | AG | CC |
| 2567 | 19315 | 268 | 71 | 23 | TC | AC | AG | CG | TC | CC | AT | AG | CG |
| 2569 | 21379 | 282 | 38 | 17 | CC | AC | AG | CG | TC | TC | AT | AG | CG |
| 2572 | 20280 | 284 | 50 | 13 | CC | AC | GG | CG | TC | CC | AT | AG | CG |
| 2574 | 18374 | 270 | 41 | 30 | CC | AA | AG | CG | TC | TC | AA | AA | CC |
| 2575 | 19903 | 291 | 46 | 9 | CC | AA | AG | CC | CC | CC | AA | AA | CC |
| 2576 | 20945 | 286 | 48 | 13 | TC | AA | AG | CC | CC | CC | AA | AA | CC |
| 2577 | 18756 | 291 | 50 | 8 | CC | AA | AG | CC | CC | CC | AA | AA | CC |
| 2586 | 21676 | 251 | 62 | 38 | TC | AA | AA | CC | CC | CC | AA | AA | CC |
| 2895 | 21097 | 249 | 65 | 38 | CC | AA | AA | CC | CC | TT | AA | AA | CC |
| 2896 | 17728 | 251 | 100 | 39 | TC | AC | AG | CG | TC | CC | AA | AG | CG |
| 2902 | 21482 | 267 | 86 | 31 | TT | AA | AA | CC | CC | CC | AA | AA | CC |
| 2906 | 20402 | 273 | 98 | 22 | CC | AA | GG | CG | CC | CC | AA | AA | CC |
| 2907 | 21414 | 247 | 97 | 45 | CC | CC | GG | CC | TT | CC | AA | GG | CC |
| 2912 | 20221 | 256 | 72 | 36 | TC | AA | AA | CC | CC | TC | AA | AA | CC |
| 2915 | 18789 | 250 | 64 | 41 | CC | AA | AG | CC | TC | TT | AA | AA | CC |
| 2918 | 21890 | 239 | 95 | 45 | TC | AA | AG | CC | CC | CC | AA | AA | GG |
| 2919 | 17389 | 252 | 131 | 46 | TT | AA | AA | CC | CC | CC | AA | AA | CC |
| 2920 | 20083 | 267 | 78 | 32 | CC | CC | GG | CC | TT | CC | AA | GG | CC |
| 2921 | 15968 | 250 | 123 | 44 | TC | AA | AA | CC | CC | TC | AA | AA | CC |
| 2923 | 20135 | 254 | 116 | 45 | CC | AC | AG | CG | TC | CC | AT | AG | CG |
| 2929 | 20743 | 258 | 96 | 37 | CC | AA | AG | CC | CC | CC | AA | AA | CC |
| 2934 | 19927 | 261 | 67 | 34 | TT | AA | AA | CC | CC | CC | AA | AA | CC |
| 2937 | 17696 | 264 | 108 | 33 | CC | AA | GG | CC | CC | CC | AA | AA | CC |
| 2939 | 17020 | 262 | 80 | 30 | CC | AA | AG | CC | CC | TC | AA | AA | CC |
| 2942 | 18527 | 253 | 116 | 44 | TC | AA | AG | CC | CC | CC | AA | AA | GG |
| 2948 | 21263 | 255 | 109 | 41 | TT | AA | AA | CC | CC | TC | AA | AA | CC |
| 2950 | 18748 | 264 | 113 | 36 | TC | AA | AA | CC | CC | CC | AA | AA | CC |
| 2951 | 19019 | 257 | 90 | 32 | CC | CC | GG | CC | TT | CC | AA | GG | CC |
| 2954 | 20399 | 263 | 67 | 31 | TT | CC | GG | CC | TT | CC | AA | GG | CC |
| 2958 | 19422 | 261 | 119 | 35 | CC | CC | GG | CC | TT | CC | AA | GG | CC |
| 2959 | 20263 | 270 | 121 | 29 | TT | AA | AG | CG | TC | TT | AA | AG | CC |
| 2961 | 17334 | 251 | 98 | 40 | CC | AC | GG | CG | TC | CC | AA | AG | CG |
| 2962 | 19884 | 246 | 143 | 49 | CC | AA | AA | GG | CC | TT | TT | AA | GG |
| 2964 | 17802 | 258 | 97 | 27 | CC | CC | GG | CG | TC | TC | AA | AG | CG |
| 2965 | 17834 | 272 | 112 | 28 | CC | AC | GG | CG | TC | CC | AT | AG | GG |
| 2967 | 22098 | 258 | 137 | 41 | TT | CC | AA | CC | TT | CC | AA | GG | GG |
| 2969 | 16400 | 257 | 110 | 41 | TT | AA | AA | CC | CC | CC | AA | AA | CC |
| 2983 | 21460 | 251 | 97 | 47 | CC | CC | AA | CC | TT | CC | AA | GG | CC |
| 2985 | 20148 | 255 | 82 | 31 | CC | CC | GG | CC | TT | CC | AA | GG | CG |
| 2988 | 17435 | 255 | 106 | 38 | CC | AA | AA | CC | CC | TT | AA | AA | CC |
| 2991 | 17857 | 249 | 83 | 41 | TT | AC | AG | CG | CC | CC | AA | AA | CC |
| 3050 | 18626 | 255 | 108 | 44 | CC | CC | GG | CC | CC | CC | AA | GG | CG |
| 3051 | 18788 | 245 | 105 | 52 | CC | CC | GG | CC | TT | CC | AA | GG | CC |
| 3054 | 20693 | 247 | 86 | 49 | TC | AC | AG | CG | TC | CC | AT | AG | CG |
| 3055 | 20750 | 243 | 138 | 57 | TT | AA | AA | GG | CC | CC | TT | AA | GG |
| 3057 | 20137 | 244 | 97 | 54 | TT | AA | AG | CG | TC | CC | AA | AG | CG |
| 3058 | 19697 | 258 | 86 | 40 | CC | AA | AG | CG | TC | CC | AA | AG | CG |
| 3059 | 16709 | 273 | 102 | 25 | CC | CC | GG | CC | TT | CC | AA | GG | CC |
| 3062 | 21606 | 245 | 75 | 28 | TC | AA | AA | CG | CC | TC | AA | AA | CC |
| 3063 | 19587 | 245 | 80 | 35 | CC | AC | AG | CG | TC | CC | AT | AG | CG |
| 3065 | 20712 | 242 | 136 | 53 | TT | AA | AA | GG | CC | TC | TT | AA | GG |
| 3066 | 18549 | 268 | 96 | 32 | TC | AA | AA | CC | CC | TC | AA | AA | CC |
| 3067 | 22048 | 237 | 125 | 58 | TT | AA | AG | CG | TC | CC | AA | AA | CG |
| 3068 | 20574 | 266 | 61 | 25 | CC | CC | AA | CC | TT | CC | AA | GG | CC |
| 3073 | 19304 | 255 | 75 | 40 | TC | CC | GG | CC | TC | CC | AA | GG | CC |
| 3074 | 17438 | 266 | 67 | 30 | CC | CC | AA | CC | TT | CC | AA | GG | CC |
| 3075 | 16891 | 248 | 96 | 48 | CC | CC | GG | CC | TT | CC | AA | GG | CC |
| 3083 | 15966 | 254 | 129 | 44 | CC | CC | GG | CC | TT | CC | AA | GG | CG |
| 3084 | 16291 | 249 | 80 | 45 | CC | AA | AG | CC | CC | TC | AA | AA | GG |
| 3085 | 17700 | 269 | 96 | 31 | TC | AA | AA | CC | CC | TC | AA | AA | CC |
| 3089 | 20454 | 268 | 84 | 31 | CC | AA | AG | CC | CC | CC | AA | AA | CC |
| 3092 | 21226 | 260 | 61 | 40 | CC | AC | AG | CG | TC | TC | AT | AG | CG |
| 3093 | 17801 | 270 | 99 | 24 | CC | AC | AG | CG | TC | CC | AT | AG | CC |
| 3094 | 19485 | 246 | 112 | 55 | TC | AA | AA | CC | CC | CC | AA | AA | CC |
| 3096 | 17730 | 252 | 103 | 47 | CC | AA | AG | CG | TC | CC | AA | AA | CG |
| 3099 | 21230 | 249 | 107 | 46 | TT | AA | AG | CG | TC | CC | AA | AA | CC |
| 3109 | 20967 | 300 | 126 | 0 | TT | AA | AA | CC | CC | CC | AA | AA | CC |
| 3110 | 20934 | 252 | 105 | 41 | TT | AA | AG | CG | TC | CC | AA | AG | CC |
| 3111 | 20900 | 263 | 68 | 31 | TT | AA | AA | CC | CC | CC | AA | AA | CC |
| 3113 | 16849 | 260 | 99 | 34 | CC | AA | AG | CC | CC | TC | AA | AA | CC |
| 3117 | 20325 | 267 | 92 | 29 | TT | AC | AG | CG | TC | CC | AT | AG | CG |
| 3118 | 21554 | 248 | 137 | 51 | CC | CC | GG | CC | CC | CC | AA | GG | CG |

**Notes.**

CAGE=cage number; cx_wb=wing number; FEA = first egg age; E59W = egg number at age 59 weeks; E300D = egg number at age 300 days.
